# Mass Flux Analysis Reveals the Presence and Fate of Antimicrobials in Hospital Effluent, Wastewater Treatment Plant, and River Environments

**DOI:** 10.3390/antibiotics15080760

**Published:** 2026-08-07

**Authors:** Takashi Azuma, Masaru Usui, Tomohiro Hasei, Tetsuya Hayashi

**Affiliations:** 1 Department of Pharmacy, Osaka Medical and Pharmaceutical University, Takatsuki 569-1094, Japan; tomohiro.hasei@ompu.ac.jp (T.H.); tetsuya.hayashi@ompu.ac.jp (T.H.); 2 Center for Infectious Disease Education and Research (CiDER), The University of Osaka, Suita 565-0871, Japan; 3 Food Microbiology and Food Safety, Department of Health and Environmental Sciences, School of Veterinary Medicine, Rakuno Gakuen University, Ebetsu 069-8501, Japan; usuima@rakuno.ac.jp

**Keywords:** antimicrobials, mass flux, hospital wastewater, wastewater treatment plants (WWTPs), river water, environmental fate, antimicrobial resistance (AMR)

## Abstract

Background: Hospitals are recognized as important point sources of antimicrobials; however, their contributions to antimicrobial contamination at the watershed scale remain poorly understood. Methods: Seasonal monitoring was conducted between December 2024 and May 2026 at two hospitals, a wastewater treatment plant (WWTP), and receiving river waters in the Yodo River Basin, Japan. Seventeen antimicrobials were quantified, and concentration profiles and mass fluxes were integrated to evaluate source contributions. Results: Ampicillin, levofloxacin, azithromycin, clarithromycin, and vancomycin were the predominant antimicrobials. Hospital wastewater was identified as the dominant source of ampicillin and vancomycin, whereas levofloxacin and clarithromycin were largely influenced by diffuse watershed inputs. Several antimicrobials persisted through wastewater treatment and were continuously detected in WWTP effluent and downstream river waters. Conclusions: This watershed-scale assessment demonstrates that antimicrobial contamination is driven by both hospital-derived point sources and broader watershed inputs. These findings provide a scientific basis for integrated hospital wastewater and WWTP management to mitigate environmental antimicrobial resistance (AMR) within the One Health framework.

## 1. Introduction

The emergence and global dissemination of antimicrobial resistance (AMR) has become one of the most serious public health challenges of the twenty-first century [1]. The burden of AMR continues to increase worldwide, with recent global assessments estimating that antimicrobial-resistant bacterial infections were directly responsible for approximately 1.27 million deaths in 2019 and are projected to cause more than 8 million deaths annually by 2050 if effective interventions are not implemented [2]. Consequently, AMR has been recognized as a critical global health issue requiring coordinated actions across human, animal, and environmental sectors under the One Health framework [3].

Although considerable progress has been made in understanding AMR in clinical and veterinary settings, the environmental dimension remains comparatively underexplored [4]. Increasing evidence indicates that aquatic environments act not only as reservoirs but also as potential hotspots for the persistence, dissemination, and evolution of antimicrobial resistance [5]. Antimicrobials discharged into aquatic environments may exert continuous selective pressure on environmental microorganisms, thereby facilitating the emergence and maintenance of antimicrobial-resistant bacteria and resistance genes [6]. Therefore, understanding the occurrence and environmental fate of antimicrobials in wastewater systems has become an essential component of One Health-based AMR mitigation strategies [7].

Following administration to humans and animals, antimicrobials are incompletely metabolized and are excreted into sewer systems through urine and feces or enter wastewater through the disposal of unused pharmaceuticals. These compounds are subsequently transported to wastewater treatment plants (WWTPs), where conventional biological treatment processes are primarily designed to remove organic matter rather than pharmaceutical contaminants [8]. Consequently, numerous antimicrobials have been detected in WWTP influent, treated wastewater, and receiving surface waters worldwide. The persistence of these compounds in aquatic environments has raised concerns regarding their ecological impacts as well as their potential role in promoting environmental AMR [9].

Hospitals are recognized as important point sources of antimicrobial discharge because of their intensive antimicrobial consumption and the continuous release of antimicrobial-containing wastewater. Previous studies have consistently reported substantially higher antimicrobial concentrations in hospital wastewater than in municipal wastewater [10]. Nevertheless, investigations focusing on hospital wastewater remain limited compared with studies of WWTPs and receiving waters. Moreover, most published studies have investigated only a single hospital and have primarily reported concentration-based occurrence data, providing limited information regarding pollution loads, source contributions, and watershed-scale environmental transport [11]. As a result, the relative contribution of hospital wastewater to antimicrobial contamination in downstream wastewater systems and aquatic environments remains insufficiently understood [12].

Our research group has previously characterized the occurrence of antimicrobials in wastewater discharged from hospitals to municipal sewer systems in urban areas of Japan [13]. However, because these investigations were limited to a single hospital, they were unable to comprehensively evaluate the environmental contribution of hospital wastewater within an entire urban watershed. Simultaneous investigations involving multiple hospitals together with the associated WWTP and receiving river are therefore required to quantify pollution loads, identify source contributions, and clarify the environmental fate of antimicrobials throughout the wastewater continuum.

In the present study, we conducted a seasonal watershed-scale investigation between December 2024 and May 2026 in the Yodo River basin, one of the largest and most important urban watersheds in Japan [14]. Wastewater samples were collected from two general hospitals of different sizes, the WWTP receiving their wastewater, and the receiving river downstream of the WWTP discharge. Seventeen antimicrobials representing five major antimicrobial classes were quantified to characterize their occurrence and distribution throughout the wastewater pathway. Furthermore, mass flux analysis was employed to quantify antimicrobial pollution loads and to estimate (i) the contribution of hospital wastewater to antimicrobial loads entering the WWTP and (ii) the contribution of WWTP effluent to antimicrobial contamination in the receiving river. To our knowledge, this is the first study to combine these specific site types within a single urban watershed using a mass flux-based approach. By integrating concentration profiles, pollution loads, source contributions, and temporal variations, this study provides new insights into the linkage between healthcare activities and the aquatic environment, advances our understanding of antimicrobial dissemination within urban watersheds from a One Health perspective, and provides a scientific basis for future hospital wastewater management and watershed-scale strategies to mitigate environmental antimicrobial pollution.

## 2. Materials and Methods

### 2.1. Chemicals and Reagents

Seventeen antimicrobials representing five major classes were selected for analysis: *β*-lactams (ampicillin, benzylpenicillin, cefdinir, cefpodoxime, cefpodoxime proxetil, and ceftiofur), new quinolones (ciprofloxacin, enrofloxacin, and levofloxacin), macrolides (azithromycin and clarithromycin), tetracyclines (chlortetracycline, doxycycline, minocycline, oxytetracycline, and tetracycline), and the glycopeptide vancomycin. These compounds were selected based on previous reports describing their occurrence and detection frequency in hospital wastewater, WWTPs, and river water in Japan and other regions, as well as their clinical usage patterns in Japan [15,16,17,18]. The physicochemical properties of the target antimicrobials are summarized in Table 1. Analytical standards with purity > 98% were obtained from Cayman Chemical (Ann Arbor, MI, USA), LKT Laboratories (St. Paul, MN, USA), Santa Cruz Biotechnology (Santa Cruz, CA, USA), Tokyo Chemical Industry (Tokyo, Japan), and Toronto Research Chemicals (Toronto, ON, Canada). Individual stock solutions (10 mg/L) were prepared in methanol according to the purity of each compound and stored at −20 °C until use. Ultrapure water (18.2 MΩ·cm) produced by a Milli-Q purification system (MilliporeSigma, Watford, UK) was used for all aqueous solutions. LC–MS-grade methanol, acetone, and formic acid, as well as reagent-grade ascorbic acid and hydrochloric acid, were purchased from FUJIFILM Wako Pure Chemical Corporation (Osaka, Japan).

### 2.2. Survey of Hospital Wastewater, Wastewater Treatment Plant, and River Water

The survey was conducted in the Yodo River Basin, Japan, which has a population of approximately 17 million (about 14% of the national population) [14,20]. To investigate the distribution of antimicrobials in environmental waters, six types of water samples were collected: two hospital effluents, WWTP influent, WWTP secondary effluent, final WWTP effluent, and two river water samples. Hospital effluents were obtained from two hospitals treating 700 (hospital 1) and 1200 patients (hospital 2) per day. These effluents are discharged into the municipal sewerage system serving a population of 420,000 and subsequently conveyed to the WWTP. Untreated influent wastewater was collected at the WWTP inlet. Wastewater is treated using a conventional activated sludge (CAS) process to produce secondary effluent, followed by additional anoxic/oxic (AO) treatment and final chlorination disinfection (0.9 mg/L NaClO for 15 min). River water samples (river 1) were collected from the main stream of the Yodo River, approximately 12 km upstream of the downstream site, where the sampling point serves as a major drinking water source supplying the study area and surrounding regions. Further, river water (river 2) was sampled at a site located 1 km downstream of the WWTP discharge point on Kanzaki River, a tributary of the Yodo River. During the study period, the annual mean biochemical oxygen demand (BOD) of river 1 was 1.0 ± 0.1 mg/L, whereas that of river 2 was 3.1 ± 0.4 mg/L.

Sampling was conducted across four seasons during 2024–2026 on the following dates: December 19 (winter), March 21 (spring), June 9 (summer), September 10 (autumn), November 19 (winter), February 4 (winter), and May 26 (spring). To minimize dilution effects from precipitation, sampling was performed on rain-free days when no rainfall (<1 mm) had been recorded during the two days preceding sampling [21]. No major infectious disease outbreaks were reported during the sampling period [22].

Water samples were collected using a 300 mL stainless-steel pail sampler, following the Guidelines for Collecting Environmental Water issued by the Ministry of Land, Infrastructure, Transport and Tourism, Japan [23]. Samples of hospital effluent, WWTP influent, WWTP secondary effluent, WWTP effluent, and river water were transferred to sterilized glass bottles. Due to regulatory restrictions, installation of automatic composite samplers at hospital and WWTP facilities was not permitted, and deployment of monitoring equipment along the river channel was legally restricted. Therefore, manual grab sampling was conducted at fixed intervals as described previously [24]. All samples were transported to the laboratory within 2 h in a cooled container, stored at 4 °C in the dark, and processed within 24 h of collection.

### 2.3. Analytical Procedures for Antimicrobials in the Environmental Water

Antimicrobials in water samples were quantified using solid-phase extraction (SPE) followed by ultra-performance liquid chromatography–tandem mass spectrometry (UPLC–MS/MS) based on previously reported procedures [25]. Briefly, 10 mL of water samples from hospital effluent and WWTP influent, and 30 mL of water samples from WWTP secondary effluent and river water samples were filtered through a glass fiber filter (GF/B, 1 μm pore size; Whatman, Maidstone, UK). The filtrate was then subjected to SPE using OASIS HLB cartridges (200 mg, 6 mL; Waters Corp., Milford, MA, USA), which were preconditioned with 3 mL methanol followed by 3 mL Milli-Q water adjusted to pH 3 with 1 N HCl. Samples were loaded at approximately 1 mL/min. After loading, the cartridges were rinsed with 6 mL of Milli-Q water (pH 3) and dried under vacuum. Target compounds were eluted with 6 mL of acetone–methanol (1:1, *v*/*v*), and the eluates were evaporated to dryness under a gentle nitrogen stream at 37 °C. The residues were reconstituted in 200 μL of 0.1% formic acid in methanol/water (90:10, *v*/*v*), and a 10 μL aliquot was injected into the UPLC–MS/MS system.

Chromatographic separation was performed using a UPLC BEH C_18_ column (2.1 mm × 100 mm, 1.7 μm; Waters Corp.) coupled to a triple quadrupole mass spectrometer (Waters Corp.). Instrument operation and data acquisition were carried out using MassLynx 4.1 software (Waters Corp.). Matrix effects and procedural losses were evaluated by comparing spiked and blank samples and corrected by subtracting background signals from those of fortified samples [26]. Recovery efficiencies were calculated by comparing responses from spiked samples with those obtained from calibration standards [27]. Limits of detection (LOD) and quantification (LOQ) were determined using signal-to-noise ratios of 3 and 10, respectively [28]. Detailed analytical performance parameters are summarized in Table S1. These are generally similar to those previously reported for pharmaceuticals in river water and wastewater samples [29]. Quantification was performed using six-point external calibration curves prepared in a solution of 0.1% formic acid in methanol/water (90:10, *v*/*v*) over a concentration range of 0.5–200 ng/mL. For concentrations exceeding this range, the concentration is calculated by measuring a 100-fold diluted solution. All target compounds exhibited excellent linearity within this range (*r*^2^ > 0.99), and a weighting factor of 1/x was applied during regression analysis. Instrument control, calibration, and data processing were conducted using MassLynx 4.1 software (Waters Corp.).

### 2.4. Calculation of Mass Loads and Hospital Effluent Contributions to WWTP Influent and the River Water Environment

The mass load value of antimicrobials was calculated by multiplying the detected concentration (ng/L) by the mean daily flow rates of the hospital, WWTP, and river water (m^3^/day) [30]. According to the flow rates on the sampling day (m^3^/day), the value at the WWTP was available, but without the corresponding values for the hospital effluent and river water. In the latter two cases, the only available data were the monthly flow rate of hospital effluent from the hospital and the daily mean river water flow rate per month provided by the Ministry of Land, Infrastructure and Transport, Osaka, Japan. The annual mean flow rates were 105 ± 3 m^3^/day and 460 ± 35 m^3^/day for hospital effluent 1 and 2, 190,000 ± 5000 m^3^/day for WWTP, 13,447,000 ± 6,341,000 m^3^/day for river 1, and 2,219,000 ± 563,000 m^3^/day for river 2. The contribution of the mass load of antimicrobials in hospital effluent to WWTP influent was calculated by dividing the individual mass load of pollutants in the hospital effluent by that in the WWTP influent. The contribution of the mass load of pollutants in WWTP effluent to river water was calculated by dividing the individual mass load of pollutants in the WWTP effluent by that of river water [31].

### 2.5. Statistical Analysis

The basic data for the tested traits were analyzed using Microsoft Excel and presented as mean values and their individual standard deviations, while other statistical analyses and graphics were performed using R software (version 4.6.1) (https://www.R-project.org/, accessed on 29 June 2026) [32]. A one-way analysis of variance (ANOVA) was performed to conduct the statistical analysis. When significant differences were observed between groups, Tukey’s honest significant difference (HSD) test was performed as a post hoc test. *p* < 0.05 was considered statistically significant. All visualizations adhered to standardized formatting and scaling procedures to ensure consistency across the comparative datasets.

## 3. Results

### 3.1. Distribution of Antimicrobial Concentrations in Hospital Effluents, WWTP, and River Waters

**Figure 1:**
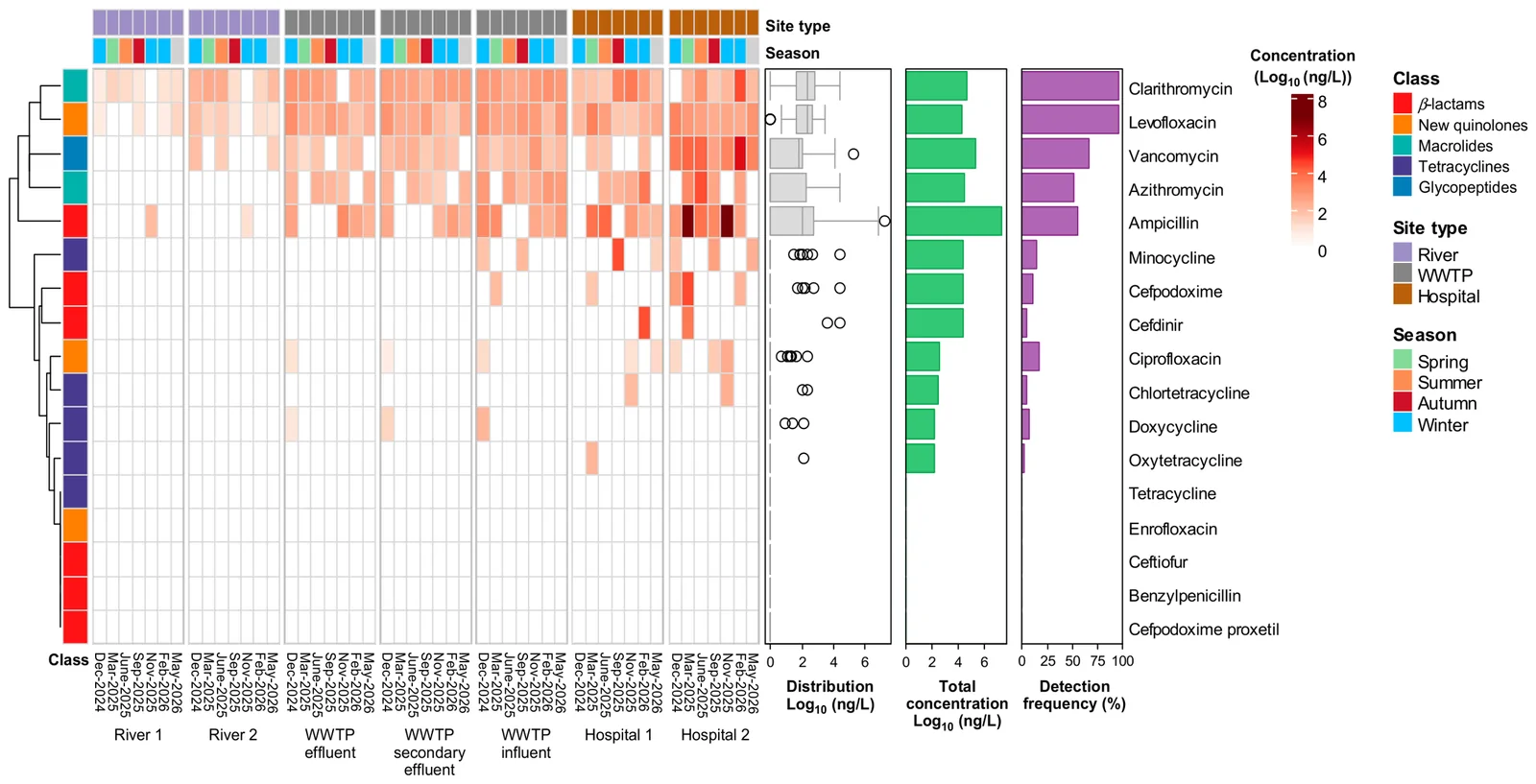
Occurrence of antimicrobials in hospital effluent, WWTP influent, WWTP secondary effluent, WWTP effluent, and river waters. The bar graph in the Figure shows the distribution of each antimicrobial over the entire study period, total concentrations, and detection frequency. (Spring: March through May, Summer: June through August, Fall: September through November, and Winter: December through February in accordance with the four seasons in Japan).

**Figure 2:**
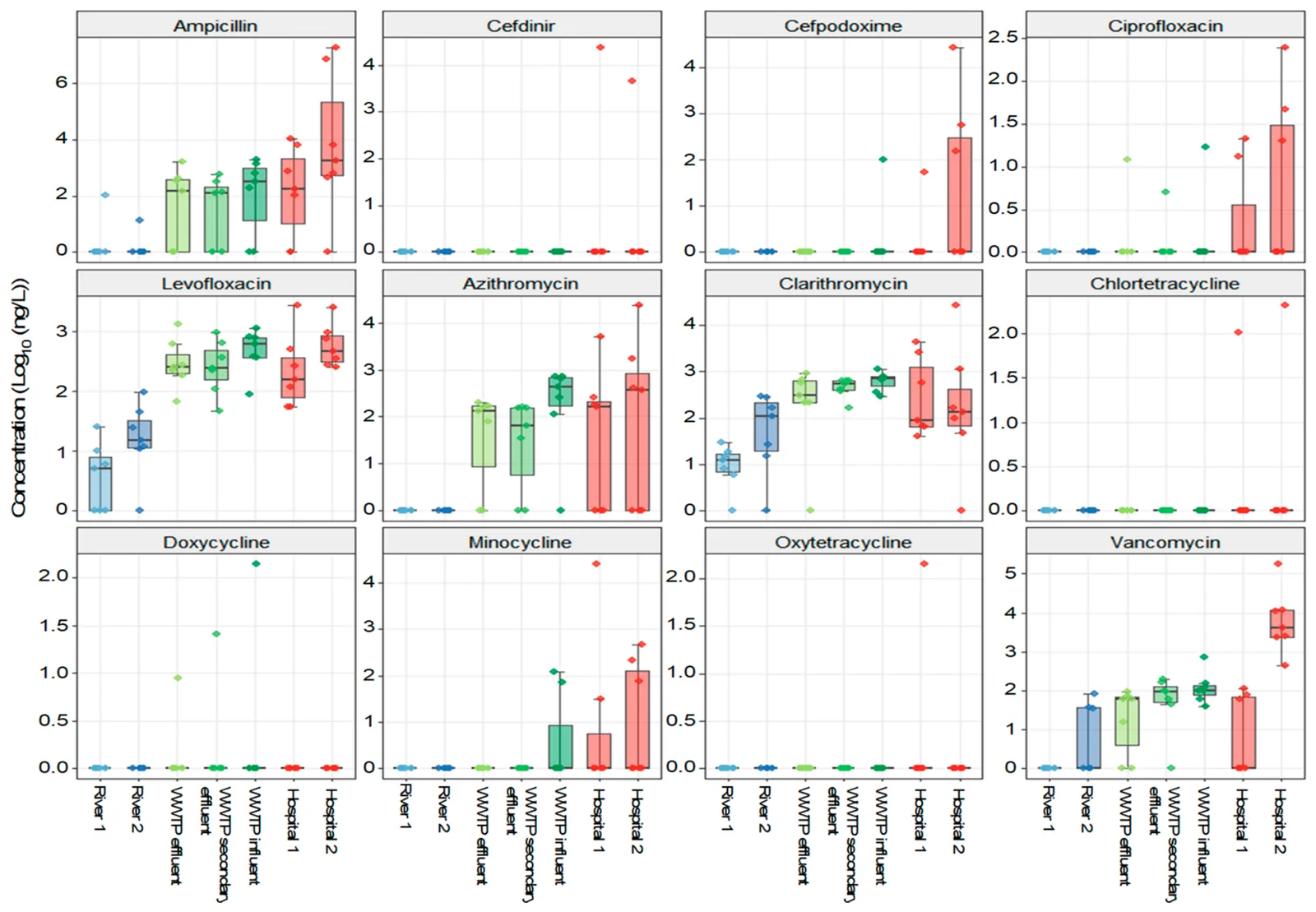
Distribution of antimicrobials in hospital effluent, WWTP influent, WWTP secondary effluent, WWTP effluent, and river waters. The vertical axis shows the concentration of the antimicrobial on a logarithmic scale.

The distribution of antimicrobial concentrations across hospital wastewaters, WWTP wastewaters, and river waters is shown in Figure 1 and Figure 2, while detailed concentration data are summarized in Table 2 and Figure S1. Among the 17 antimicrobials investigated, ampicillin, levofloxacin, azithromycin, clarithromycin, and vancomycin exhibited both high detection frequencies and high concentrations throughout the study period. In contrast, benzylpenicillin, cefdinir, cefpodoxime proxetil, ceftiofur, enrofloxacin, and tetracycline were not detected in any environmental samples.

Overall, antimicrobial concentrations were substantially higher in hospital wastewaters than in WWTP wastewaters or river waters. In hospital effluent 1, mean concentrations ranged from 36 to 3631 ng/L, with maximum concentrations between 20 and 23,875 ng/L. In hospital effluent 2, considerably higher concentrations were observed, ranging from 30 to 3,723,848 ng/L on average and reaching maximum values of 208 to 18,580,444 ng/L, representing approximately one order of magnitude higher concentrations than those measured in hospital effluent 1. These spatial differences suggest variations in antimicrobial usage patterns, wastewater handling practices, and potential removal efficiencies [33].

Among the *β*-lactams, cefdinir and cefpodoxime proxetil exhibited marked differences between the two hospitals. Cefdinir was detected at mean concentrations of 3411 ng/L and 642 ng/L in hospital effluent 1 and hospital effluent 2, respectively, whereas cefpodoxime proxetil was detected at 8 ng/L and 4047 ng/L, respectively. However, these compounds were either absent or detected only at trace levels in WWTP influent (N.D. and 15 ng/L, respectively) and were not detected in WWTP secondary effluent, WWTP effluent, or either river throughout the study period. In contrast, ampicillin remained detectable throughout the wastewater continuum. Mean concentrations reached 2589 ng/L in hospital effluent 1 and 3,723,848 ng/L in hospital effluent 2, decreasing to 636 ng/L in WWTP influent, 89 ng/L in WWTP secondary effluent, and 412 ng/L in WWTP effluent, before declining further to 15 ng/L in river 1 and 2 ng/L in river 2. The detection frequency of ampicillin also progressively decreased along the wastewater pathway, from 71 to 86% in hospital wastewaters and 71% in WWTP influent to 57% in WWTP effluent and 14% in river waters. These results suggest that hospitals are the main source of ampicillin in wastewater [34], but it tends to decrease in wastewater treatment systems and environmental water [35].

For the new quinolones, levofloxacin was consistently detected in all types of wastewater and river water, exhibiting detection frequencies of 100% throughout the wastewater system and 57–86% in river waters. Mean concentrations were 556 ng/L in hospital effluent 1, 811 ng/L in hospital effluent 2, 603 ng/L in WWTP influent, 438 ng/L in WWTP secondary effluent, and 539 ng/L in WWTP effluent, indicating persistence at several hundred ng/L even after wastewater treatment. Mean concentrations subsequently declined to 6 ng/L in river 1 and 28 ng/L in river 2. Ciprofloxacin showed a similar distribution pattern but occurred at substantially lower concentrations, generally ranging from a few to several tens of ng/L, with detection frequencies of 14–43%. Enrofloxacin was not detected in any sample. The widespread presence of levofloxacin in both hospital effluent and WWTP influent indicates contributions from both clinical and community sources, which is consistent with its extensive use in inpatient and outpatient settings [36].

Among the macrolides, azithromycin and clarithromycin were frequently detected throughout the wastewater system. Detection frequencies for azithromycin were 57% in hospital wastewaters, 86% in WWTP influent, 71% in WWTP effluent, and not detected in river waters. Corresponding frequencies for clarithromycin were 86–100%, 100%, 86%, and 86%, respectively. Mean concentrations of azithromycin were 802 ng/L and 3659 ng/L in hospital effluent 1 and hospital effluent 2, respectively, decreasing to 411 ng/L in WWTP influent, 80 ng/L in WWTP secondary effluent, and 113 ng/L in WWTP effluent, while remaining below the detection limit in river 1 but averaging 12 ng/L in river 2. Clarithromycin followed a similar trend, with mean concentrations of 1122 ng/L and 4017 ng/L in hospital effluent 1 and hospital effluent 2, respectively, 669 ng/L in WWTP influent, 433 ng/L in WWTP secondary effluent, 497 ng/L in WWTP effluent, and 126 ng/L in river 2, whereas it was not detected in river 1. The widespread presence of macrolides is likely due to their broad-spectrum activity and extensive use in inpatient and outpatient settings, which is consistent with national prescription statistics in Japan [18].

Detection frequencies of tetracyclines were generally low (approximately 10–30%). Chlortetracycline and minocycline were detected only sporadically in hospital wastewaters, with mean concentrations of 15 ng/L and 3631 ng/L in hospital effluent 1 and 30 ng/L and 107 ng/L in hospital effluent 2, respectively. Minocycline was additionally detected in WWTP influent at a mean concentration of 27 ng/L, but was not detected in WWTP secondary effluent, WWTP effluent, or river waters. Doxycycline was detected at mean concentrations of 20 ng/L in WWTP influent and 5 ng/L in WWTP effluent but was not detected in either river. These localized, heterogeneous distributions likely reflect the specialized clinical applications of tetracycline-class antimicrobials, which may be associated with particular hospital departments or prescription practices [37].

The glycopeptide vancomycin exhibited occurrence patterns comparable to those of the macrolides. Detection frequencies ranged from 43 to 100% in hospital wastewaters and reached 100% in WWTP influent, before decreasing to 71% in WWTP effluent and 0–43% in river waters. Mean concentrations were 36 ng/L in hospital effluent 1 and markedly higher (30,313 ng/L) in hospital effluent 2, subsequently declining to 183 ng/L in WWTP influent, 118 ng/L in WWTP secondary effluent, and 42 ng/L in WWTP effluent. In river waters, vancomycin was detected only in river 2 at a mean concentration of 22 ng/L and was not detected in river 1. These patterns likely reflect the targeted use of these antimicrobials in inpatient and intensive care settings, given their clinical role in treating antimicrobial-resistant infections [38].

### 3.2. Distribution of Antimicrobial Classes Across Hospital Effluents, WWTP, and River Waters

**Figure 3:**
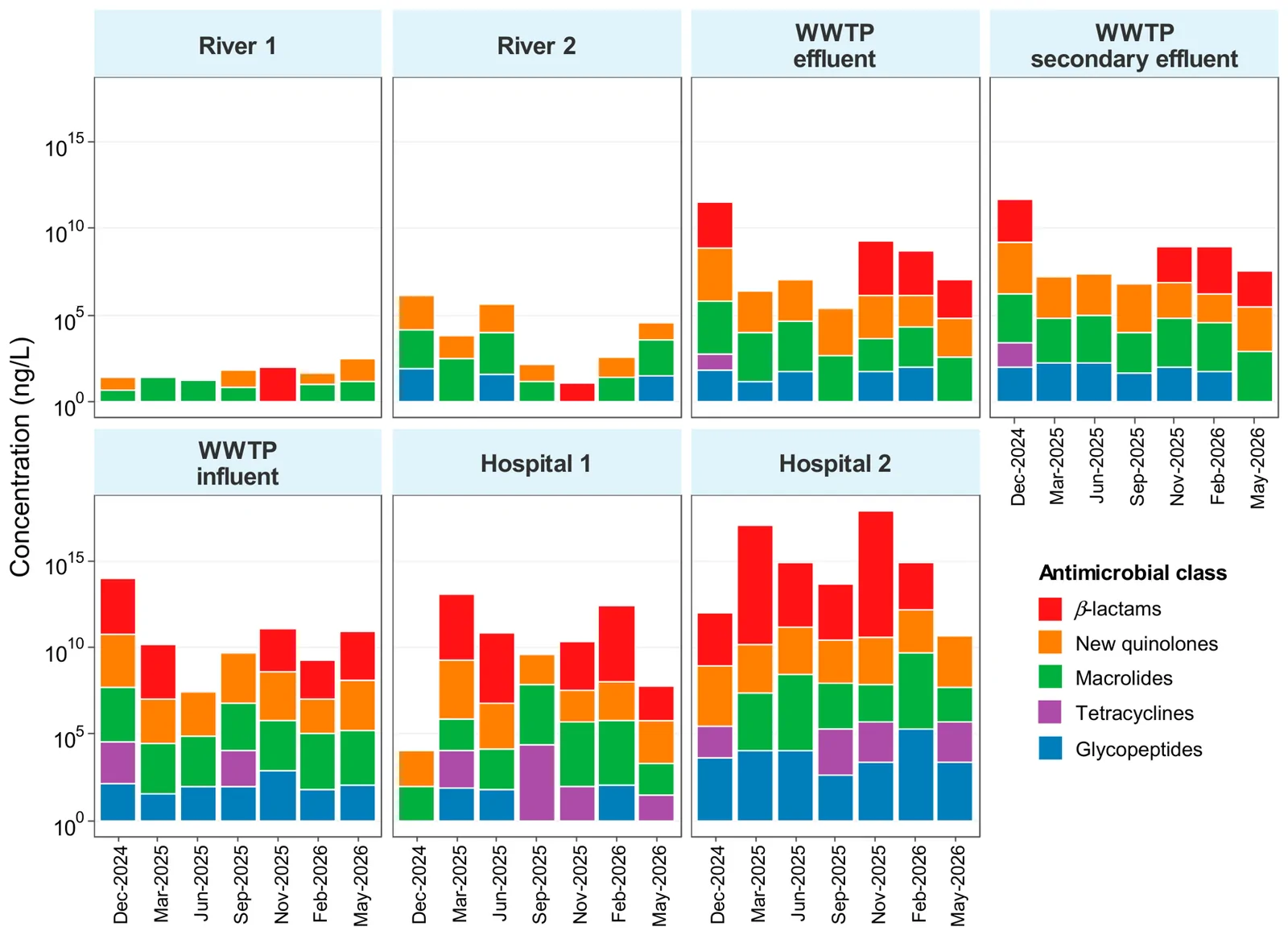
Distribution of antimicrobial classes across hospital effluents, WWTP, and river waters. The vertical axis shows the concentrations for each antimicrobial class on a logarithmic scale.

The distribution of antimicrobial classes across hospital wastewaters, WWTP wastewaters, and river waters is presented in Figure 3. Significant differences in the total concentrations of antimicrobial classes were observed between river water and WWTP effluent. (*p* < 0.05). On the other hand, statistically significant differences were not observed among WWTP secondary effluent, WWTP effluent, and hospital effluent. The highest total antimicrobial concentration was observed in hospital effluent 2, ranging from 2957 to 18,584,090 ng/L, whereas the corresponding concentrations in hospital effluent 1 ranged from 459 to 30,838 ng/L.

The antimicrobial profiles differed substantially between the two hospitals (*p* < 0.05). In hospital effluent 1, *β*-lactams and macrolides were the dominant antimicrobial classes throughout the study period. On 4 February 2026, *β*-lactams and macrolides reached 24,045 ng/L and 5594 ng/L, respectively, accounting for the majority of the total antimicrobial concentration (30,011 ng/L). A distinct compositional shift was observed on 19 November 2025, when tetracyclines reached 25,389 ng/L, representing approximately 82% of the total antimicrobial concentration (30,838 ng/L).

In contrast, hospital effluent 2 was consistently dominated by *β*-lactams and glycopeptides. Exceptionally high *β*-lactam concentrations were observed on 21 March 2025 (7,509,422 ng/L) and 19 November 2025 (18,580,444 ng/L), accounting for more than 99% of the corresponding total antimicrobial concentrations (7,523,229 and 18,584,090 ng/L, respectively). On 4 February 2026, glycopeptides and macrolides reached 180,080 ng/L and 26,964 ng/L, respectively, contributing substantially to the total antimicrobial concentration of 208,132 ng/L. The types and concentrations of antimicrobials detected in hospital wastewater are believed to be influenced by factors such as the patient population, antimicrobial usage, the wastewater system, and the timing of water sampling. The results of this study would reflect the combined effects of these factors [15].

In WWTP influent, macrolides represented the predominant antimicrobial class throughout the monitoring period, with concentrations ranging from 469 to 1833 ng/L. Total antimicrobial concentrations ranged from 1190 to 4711 ng/L, while *β*-lactams, new quinolones, and glycopeptides ranged from N.D.–1878 ng/L, 87–1166 ng/L, and 39–714 ng/L, respectively. Following wastewater treatment, macrolides and new quinolones remained the dominant antimicrobial classes in both WWTP secondary effluent and effluent. Total antimicrobial concentrations ranged from 834 to 2080 ng/L in WWTP secondary effluent and from 713 to 2889 ng/L in effluent. Concentrations of macrolides ranged from 227 to 771 ng/L in WWTP secondary effluent and from 76 to 1074 ng/L in WWTP effluent, while new quinolones ranged from 46 to 962 ng/L and 66 to 1333 ng/L, respectively. These antimicrobial classes remained detectable at concentrations of several hundred ng/L after wastewater treatment.

Significant differences were also observed between the two river sampling sites (*p* < 0.05). In river 1, total antimicrobial concentrations ranged from 10 to 105 ng/L and were primarily composed of macrolides and new quinolones. In contrast, river 2 exhibited substantially higher total antimicrobial concentrations, ranging from 12 to 357 ng/L, with maximum concentrations approximately tenfold higher than those observed in river 1. On 19 December 2024, concentrations of macrolides, new quinolones, and glycopeptides reached 162 ng/L, 97 ng/L, and 82 ng/L, respectively, resulting in a total antimicrobial concentration of 341 ng/L. Throughout the study period, macrolides consistently represented the dominant antimicrobial class in river 2, reaching a maximum concentration of 296 ng/L. The composition and total concentration of antimicrobials found in various environmental water samples suggest the potential impact of wastewater on the aquatic environments. These highlight the need for advanced wastewater treatment systems that can remove these contaminants [39].

### 3.3. Mass Fluxes and Contribution of Hospital Effluents to WWTP Influent, and Contribution of WWTP Effluent to River Water

Mass fluxes and the contributions of hospital effluents to WWTP influent are summarized in Table 3. Among all antimicrobials investigated, ampicillin exhibited by far the greatest mass flux from hospital effluents to the WWTP influent. However, substantial differences were observed between the two hospitals. The mean mass flux of ampicillin from hospital effluent 2 reached 1708 ± 3265 g/day, whereas that from hospital effluent 1 was only 0.3 ± 0.5 g/day, representing a difference of more than three orders of magnitude. Consequently, the estimated contribution of hospital effluent 2 to the maximum ampicillin load entering the WWTP was 89,408%, indicating that the calculated mass flux from this hospital substantially exceeded the measured influent load. A similar pattern was observed for cefpodoxime, for which the maximum estimated contribution reached 409%.

Among the new quinolones, levofloxacin showed a relatively high mass flux in the WWTP influent (93 ± 77 g/day). In contrast, its mean mass fluxes from hospital effluent 1 and hospital effluent 2 were only 0.1 g/day and 0.3 g/day, respectively, accounting for only 2% (maximum 6%) of the total influent load.

For the macrolides, the mean mass flux of azithromycin in the WWTP influent was 61 ± 60 g/day, with hospital effluents contributing 2% (maximum 82%) of the total load. Clarithromycin exhibited a mean influent mass flux of 106 ± 70 g/day, with hospital effluents contributing 3% (maximum 37%). For both compounds, hospital effluent 2 contributed higher mass fluxes (0.7 ± 4.0 g/day for azithromycin and 1.8 ± 4.6 g/day for clarithromycin) than hospital effluent 1 (0.1 ± 1.7 g/day and 0.1 ± 0.2 g/day, respectively).

Among the tetracyclines, only minocycline allowed estimation of the contribution from hospital effluents to the WWTP influent, with a mean contribution of 41% (maximum 81%). Oxytetracycline was detected only at trace levels in hospital effluent 1 (0.01 ± 0.01 g/day) but was not detected in the WWTP influent. Although doxycycline exhibited a mean influent mass flux of 4 ± 10 g/day, it was not detected in either hospital effluent.

For the glycopeptide vancomycin, the mean mass flux entering the WWTP influent was 31 ± 47 g/day, while the combined contribution from hospital effluents averaged 41% (range: 5–4478%). Similar to ampicillin, hospital effluent 2 showed a substantially higher mass flux (14 ± 30 g/day) than hospital effluent 1 (0.01 ± 0.01 g/day), exceeding it by more than three orders of magnitude.

Mass fluxes and the contributions of WWTP effluent to river water are summarized in Table 4. Mass fluxes from WWTP effluent to the receiving river were estimated for ampicillin, levofloxacin, clarithromycin, and vancomycin, with levofloxacin and clarithromycin exhibiting the largest riverine mass fluxes. The mean mass flux of levofloxacin was 61 ± 67 g/day in the WWTP effluent and 59 ± 76 g/day in the river, corresponding to a median contribution of 85%.

For ampicillin, the mean mass flux was 28 ± 43 g/day in the WWTP effluent and 4 ± 10 g/day in the river. Levofloxacin exhibited comparable mass fluxes in the WWTP effluent (61 ± 67 g/day) and river (59 ± 76 g/day). In contrast, clarithromycin showed a substantially higher river mass flux (245 ± 295 g/day) than that in the WWTP effluent (75 ± 46 g/day), resulting in an estimated contribution of 39%. For vancomycin, the mean mass flux was 18 ± 13 g/day in the WWTP effluent and 38 ± 71 g/day in the river, corresponding to a contribution of 10%.

In contrast, azithromycin and the tetracycline antimicrobials were not included in the river mass flux estimation because they were either not detected in the WWTP effluent or were not detected in river water during the survey period.

These results reflect the exclusive use of levofloxacin, clarithromycin, and vancomycin in human medicine, whereas ciprofloxacin is widely used in both human and veterinary contexts. This highlights the importance of distinguishing between anthropogenic and agricultural sources of antimicrobial pollution [40].

### 3.4. Temporal Variations and Distribution Patterns of Antimicrobials in Hospital and WWTP Wastewaters

**Figure 4:**
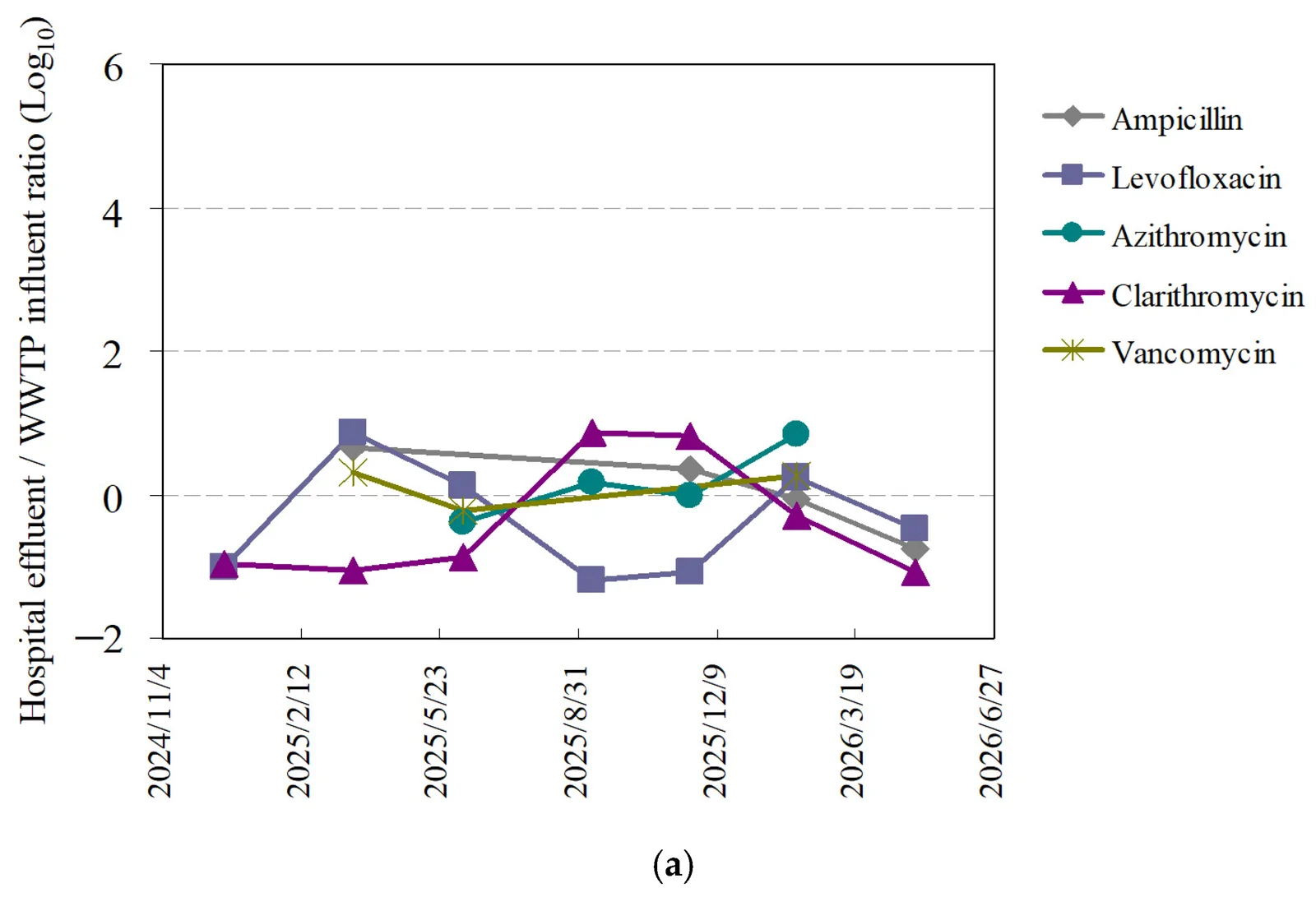

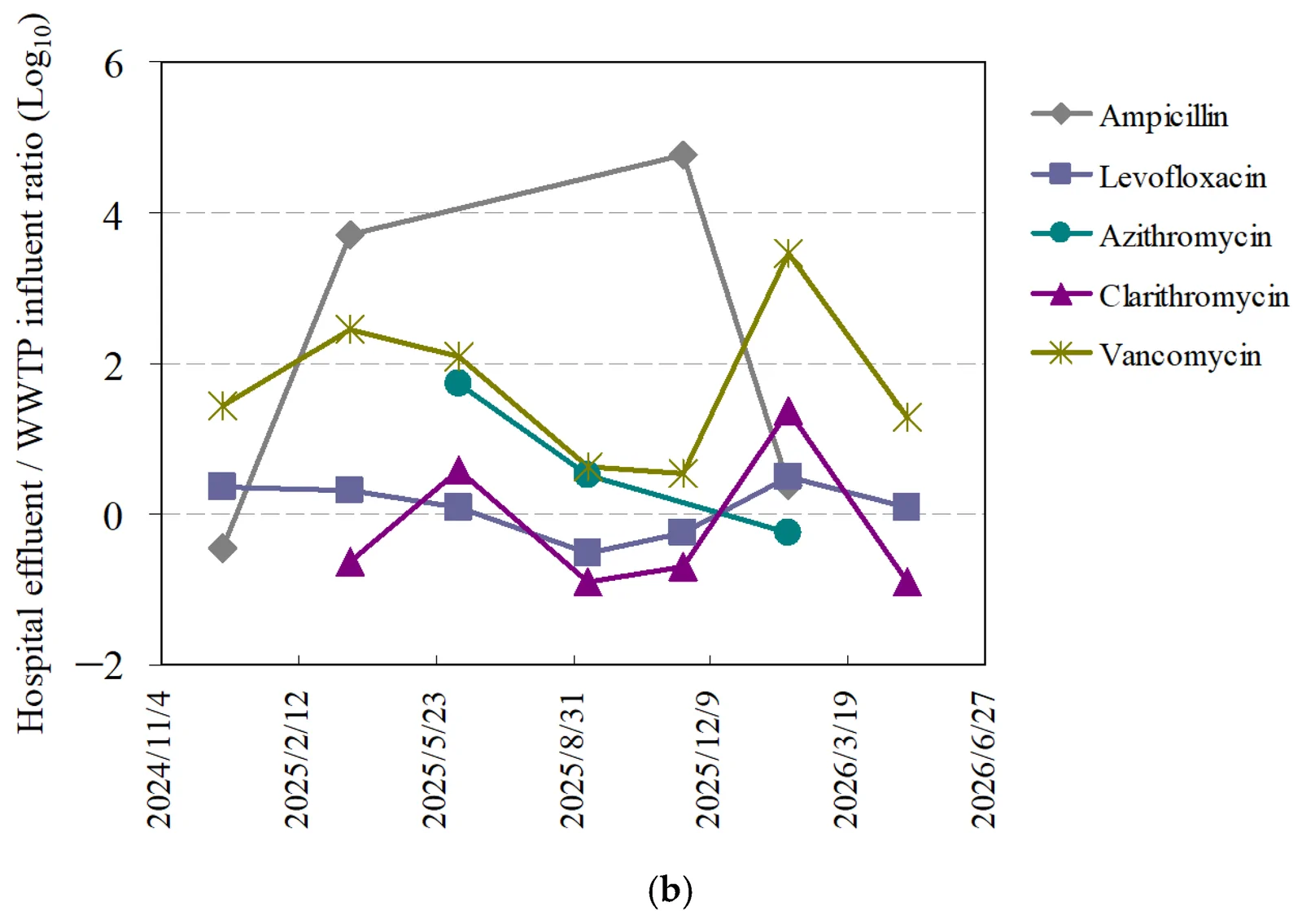
Temporal variation in the concentration ratio (hospital effluent/WWTP influent) for three representative antimicrobials (ampicillin, levofloxacin, azithromycin, clarithromycin, and vancomycin) over the sampling period from December 2024 to June 2026. The horizontal axis shows the passage of time during the drainage sampling period. ((**a**) hospital 1 and (**b**) hospital 2) Data points are displayed when an antimicrobial is detected in both locations. If an antimicrobial is not detected at either location, no data point is shown in the Figure.

The ratios of antimicrobial concentrations in hospital effluents relative to those in the WWTP influent for the five antimicrobials consistently detected in both wastewater matrices (ampicillin, levofloxacin, azithromycin, clarithromycin, and vancomycin) are presented in Figure 4.

Overall, antimicrobial concentration ratios differed markedly between the two hospitals. Hospital effluent 2 exhibited substantially higher concentration ratios than hospital effluent 1, particularly for ampicillin and vancomycin. For ampicillin, the concentration ratio in hospital effluent 2 reached a maximum of 59,052 on 19 November 2025 and 5197 on 21 March 2025, with an overall mean of 12,850 ± 25,925. The corresponding mean ratios for the other antimicrobials were 1.5 ± 1.0 (maximum 3.1) for levofloxacin, 15 ± 27 (maximum 54) for azithromycin, 4.8 ± 9.6 (maximum 24) for clarithromycin, and 489 ± 1093 (maximum 2957) for vancomycin.

In contrast, concentration ratios in hospital effluent 1 were consistently lower throughout the monitoring period. The mean ratios were 2.0 ± 1.9 (maximum 4.6) for ampicillin, 2.0 ± 2.8 (maximum 6.9) for azithromycin, 2.1 ± 3.3 (maximum 7.3) for clarithromycin, 1.6 ± 2.6 (maximum 7.4) for levofloxacin, and 1.1 ± 1.0 (maximum 2.0) for vancomycin. Temporal fluctuations in these ratios may reflect variations in patient load, treatment regimens, or institutional antimicrobial stewardship practices [41].

For hospital effluent 1, the mean concentration ratios exceeded 1 for all five antimicrobials, indicating that antimicrobial concentrations in the hospital effluent were generally comparable to or higher than those measured in the WWTP influent throughout the monitoring period. In comparison, hospital effluent 2 exhibited substantially higher concentration ratios than hospital effluent 1 for all antimicrobials except levofloxacin. The largest differences were observed for ampicillin and vancomycin, whose concentration ratios reached several thousand to tens of thousands during specific sampling events. By contrast, levofloxacin exhibited similar mean concentration ratios in both hospitals (approximately 1.5), indicating relatively small differences between hospital effluent and WWTP influent concentrations. Significant variability in usage across hospital and outpatient settings is implied by these patterns [42].

## 4. Discussion

In this study, a watershed-scale investigation was conducted across the Yodo River basins to comprehensively evaluate antimicrobial occurrence in hospital effluents, WWTP wastewaters, and river waters by integrating concentration distributions, antimicrobial composition, mass fluxes, and source contributions. The results demonstrated that the environmental behavior of antimicrobials differed markedly among individual compounds and that hospital effluents constituted an important point source for selected antimicrobials, with their influence propagating throughout the wastewater treatment system and ultimately reaching the receiving river.

From the perspective of concentration distributions, hospital effluents consistently exhibited substantially higher antimicrobial concentrations than WWTP wastewaters and river waters. Particularly high concentrations of ampicillin and vancomycin were observed in hospital effluent 2, whereas antimicrobial profiles differed considerably between the two hospitals. These findings likely reflect differences in hospital characteristics, including hospital size, clinical departments, patient populations, and antimicrobial prescribing practices, all of which can influence the composition and magnitude of antimicrobial discharge into wastewater [43]. The pronounced inter-hospital variability further indicates that hospital effluents should not be regarded as a homogeneous wastewater source but rather as highly heterogeneous point sources with distinct emission characteristics [44].

Comparison of antimicrobial concentration ratios between hospital effluents and WWTP influent further highlighted compound-specific source contributions. Extremely high concentration ratios were observed for ampicillin and vancomycin, reaching several thousand to tens of thousands and several hundred to several thousand, respectively, indicating that hospital effluents represented dominant sources of these antimicrobials entering the sewer system during specific sampling events. In fact, given that previous studies have reported notably high levels of these antibiotics in rivers near the study watershed [45], the findings of this study are consistent with and complementary to those reports. In contrast, the concentration ratios of levofloxacin and clarithromycin remained close to unity throughout the monitoring period, suggesting that their occurrence in WWTP influent was influenced by substantial inputs from non-hospital sources, including domestic wastewater [36]. These observations demonstrate that the relative importance of hospital-derived emissions varies considerably among antimicrobials and depends strongly on compound-specific usage patterns and source distributions [46].

Although antimicrobial concentrations generally decreased during wastewater treatment, several compounds remained detectable throughout the treatment process. In particular, levofloxacin, clarithromycin, and azithromycin were consistently detected in both WWTP secondary effluent and WWTP effluent, typically at concentrations ranging from several tens to several hundreds of ng/L. Moreover, antimicrobial class analysis showed that macrolides and new quinolones remained the predominant classes in WWTP effluent, indicating that these compounds were incompletely removed during conventional wastewater treatment [47]. The factors contributing to the poor degradability of these antimicrobials have not yet been fully elucidated. However, it may be due to the absence of highly reactive conjugated double bonds in their chemical structures [48]. Further, in order to gain a more comprehensive understanding of the environmental fate of fluoroquinolone and tetracycline antimicrobials in water, it is important to consider their adsorption onto activated sludge during the biological treatment process at WWTPs [49]. Consequently, WWTPs function as continuous pathways through which residual antimicrobials are discharged into aquatic environments, thereby contributing to their long-term environmental occurrence [50].

The mass flux analysis provided additional insights that could not be obtained from concentration data alone [51]. Contributions of hospital effluents to WWTP influent varied substantially among antimicrobials. Ampicillin and vancomycin exhibited exceptionally high contribution rates, confirming that hospitals functioned as dominant point sources for these compounds. Conversely, hospital contributions to the total mass fluxes of levofloxacin and clarithromycin were relatively small despite their high occurrence in WWTP influent, indicating that diffuse inputs, particularly domestic wastewater, constituted the primary sources of these antimicrobials. This interpretation is consistent with previous international studies that estimated that hospital effluent may contribute between 0.01% and 59% of pharmaceuticals [52,53], and 15% to 71% of X-ray contrasts [54] to WWTPs. Expanding hospital sampling and conducting continuous monitoring are essential for a more comprehensive understanding of hospital contributions to catchment-scale antimicrobial pollution. These findings highlight the importance of combining concentration measurements with mass flux analysis to distinguish dominant emission sources and to accurately characterize antimicrobial transport within urban watersheds [55].

Analysis of mass fluxes from WWTP effluent to the receiving river further demonstrated that levofloxacin and clarithromycin were among the major contributors to riverine antimicrobial loads. These results are considered valid when compared to the concentrations of antimicrobials detected in river water in this study, as well as to the results of previous studies [49]. River 2, located downstream of the WWTP discharge point, consistently exhibited higher total antimicrobial concentrations than river 1, together with elevated concentrations of macrolides, new quinolones, and glycopeptides, supporting the importance of WWTP effluent as a major source of antimicrobial contamination in the receiving water. However, for clarithromycin and vancomycin, riverine mass fluxes occasionally exceeded those discharged from the investigated WWTP, suggesting the presence of additional upstream or local emission sources within the watershed. Furthermore, it is believed that these antimicrobials are resistant to photolysis by sunlight and biodegradation by microorganisms as they flow through rivers, causing them to persist in the aquatic environment for long periods of time and reach downstream areas without attenuating [56]. These findings indicate that antimicrobial contamination in urban river systems is governed not only by WWTP discharges but also by multiple point and diffuse sources distributed throughout the catchment. Accordingly, the environmental fate of antimicrobials should be evaluated from a watershed perspective that links emission sources, wastewater conveyance, treatment processes, and receiving waters as an integrated system [57]. On the other hand, regarding the phenomenon in which the contribution of the load exceeds 100%, attenuation in the environmental water, in addition to the sampling method, may also be a factor. For instance, ampicillin is highly susceptible to hydrolysis; therefore, its concentration would fluctuate as it flows from hospitals to wastewater treatment plants via the sewer system [58]. In addition, when it comes to seasonal sampling, it’s important to consider the possibility that the results could be affected by uneven sampling density. Since the present survey for this study was not conducted at an equal frequency across all seasons, studies aimed at improving sampling methods and equipment are important [59]. Furthermore, this study suggests the existence of other pathways that have not yet been thoroughly examined, such as the flow of wastewater from agricultural areas into WWTPs. It is crucial to expand the scope of future studies to include a wider variety of facilities in order to conduct a more detailed investigation [60].

Overall, the present study demonstrates that effective management of antimicrobial contamination in urban watersheds requires an integrated strategy extending beyond hospital wastewater control alone [61]. While targeted management of hospital effluents may substantially reduce emissions of selected antimicrobials such as ampicillin and vancomycin, reducing the environmental burden of more widely used compounds, including levofloxacin and clarithromycin, will additionally require improvements in WWTP treatment performance together with watershed-scale source control. This study provides new insights into the transport and fate of antimicrobials from hospitals to receiving rivers within highly urbanized watersheds. The present results provide a scientific basis for evaluating the environmental risks of AMR and for integrated watershed management in areas with dense concentrations of healthcare facilities and municipal wastewater infrastructures [62].

The findings of this study have several important implications for antimicrobial management in urban watersheds. First, hospital-specific control measures should be prioritized for antimicrobials such as ampicillin and vancomycin, for which hospital effluents represented dominant point sources to the WWTP. Second, the continuous occurrence of levofloxacin, clarithromycin, and azithromycin in WWTP effluents indicates that conventional wastewater treatment alone is insufficient, highlighting the need for advanced treatment technologies capable of improving antimicrobial removal. Third, the relatively limited hospital contribution to several compounds suggests that effective pollution control should also include domestic wastewater and other diffuse sources through watershed-scale source management. Integrating hospital wastewater control, WWTP optimization, and catchment-wide monitoring within a One Health framework will be essential for reducing environmental antimicrobial contamination and mitigating the dissemination of antimicrobial resistance in aquatic ecosystems [63].

## 5. Limitations

Clarifying the environmental impact of antibiotics inherent in medical institutions and wastewater treatment plants at the watershed scale is important for deepening our understanding of environmental AMR. Further improvements to these aspects of investigation and analysis should be considered in the future.

The sampling method used in this study is a limitation of the results. Manual grab sampling was used instead of 24-h flow-proportional composite samples, so the results may differ from those averaged over a single day. Additionally, the mass flux analysis uses annual and monthly average flow rates to calculate daily mass loads, which introduces a methodological limitation: daily mass flux values may contain errors. To address these issues, it is important to increase the sampling frequency and improve the water sampling equipment [64].

Second, prior to study initiation, all participating hospitals required the exclusion of any information that could enable institutional identification, including regional location, bed capacity, prescription profiles, and wastewater flow volumes. All reported data therefore fully comply with research ethics and confidentiality agreements. Consequently, direct analyses linking antimicrobial occurrence to hospital-specific clinical practices or facility characteristics were not feasible in the present study. Future multi-center investigations conducted under harmonized data-sharing frameworks, at both national and international scales, are expected to expand the available evidence and enable more detailed comparative assessments.

Addressing these methodological constraints represents a key priority and an important direction for future research in environmental antimicrobial studies. Our results support the need for further conclusive research that considers experimental, technical, and regional customs, bias, and unknown factors.

## 6. Conclusions

This study comprehensively evaluated the concentrations, mass fluxes, and source contributions of antimicrobials across hospital effluents, a WWTP, and receiving river waters within the Yodo River watershed, Japan. Ampicillin, levofloxacin, azithromycin, clarithromycin, and vancomycin were identified as the predominant antimicrobials throughout the wastewater system, while hospital effluent 2 exhibited exceptionally high concentrations of ampicillin and vancomycin.

Mass flux analysis demonstrated that hospital wastewaters were major point sources of ampicillin and vancomycin. In contrast, the relatively limited contributions of hospital effluents to levofloxacin and clarithromycin indicated that diffuse sources, including municipal wastewater, played a more substantial role in determining their watershed-scale loads. Furthermore, levofloxacin, clarithromycin, and azithromycin persisted throughout the wastewater treatment process and were continuously discharged via WWTP effluent, highlighting the important role of WWTPs as pathways for antimicrobial release into urban river systems. The observation that riverine loads of several antimicrobials exceeded those discharged from the investigated WWTP further suggests that multiple point and non-point sources collectively shape antimicrobial contamination at the watershed scale.

Furthermore, the findings of this study suggest the effectiveness of developing tailored countermeasures for each class of antimicrobial. For instance, advanced wastewater treatment is an effective method for removing macrolides and quinolones, which persist in environmental water at high concentrations. In contrast, on-site degradation treatment within hospital facilities is an effective method for removing glycopeptides, which are used specifically in hospitals. These measures balance costs and benefits according to the required reduction levels and scale and are believed to be effective.

Overall, this study provides the first watershed-scale assessment integrating hospital effluents, WWTPs, and receiving rivers to quantitatively elucidate the environmental fate and source contributions of antimicrobials in an urban catchment. These findings provide a scientific basis for developing integrated watershed management strategies that combine hospital wastewater control with improved WWTP performance, thereby supporting environmental AMR risk mitigation and advancing the implementation of the One Health approach in urban aquatic environments.

## Figures and Tables

**Table 1:** Chemical structures and properties of the antimicrobials [19].

Classification	CAS Registry Number	Antimicrobials	Molecular Formula	Molecular Mass (g/mol)	Structure	p*K*_a_	Log*P*
*β*-lactams	69-53-4	Ampicillin	C_16_H_19_N_3_O_4_S	349.4	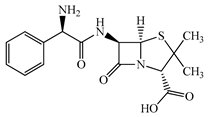	2.4	1.4
	61-33-6	Benzylpenicillin	C_16_H_18_N_2_O_4_S	334.4	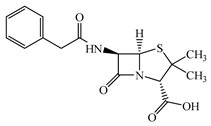	2.5	1.7
	91832-40-5	Cefdinir	C_14_H_13_N_5_O_5_S_2_	395.4	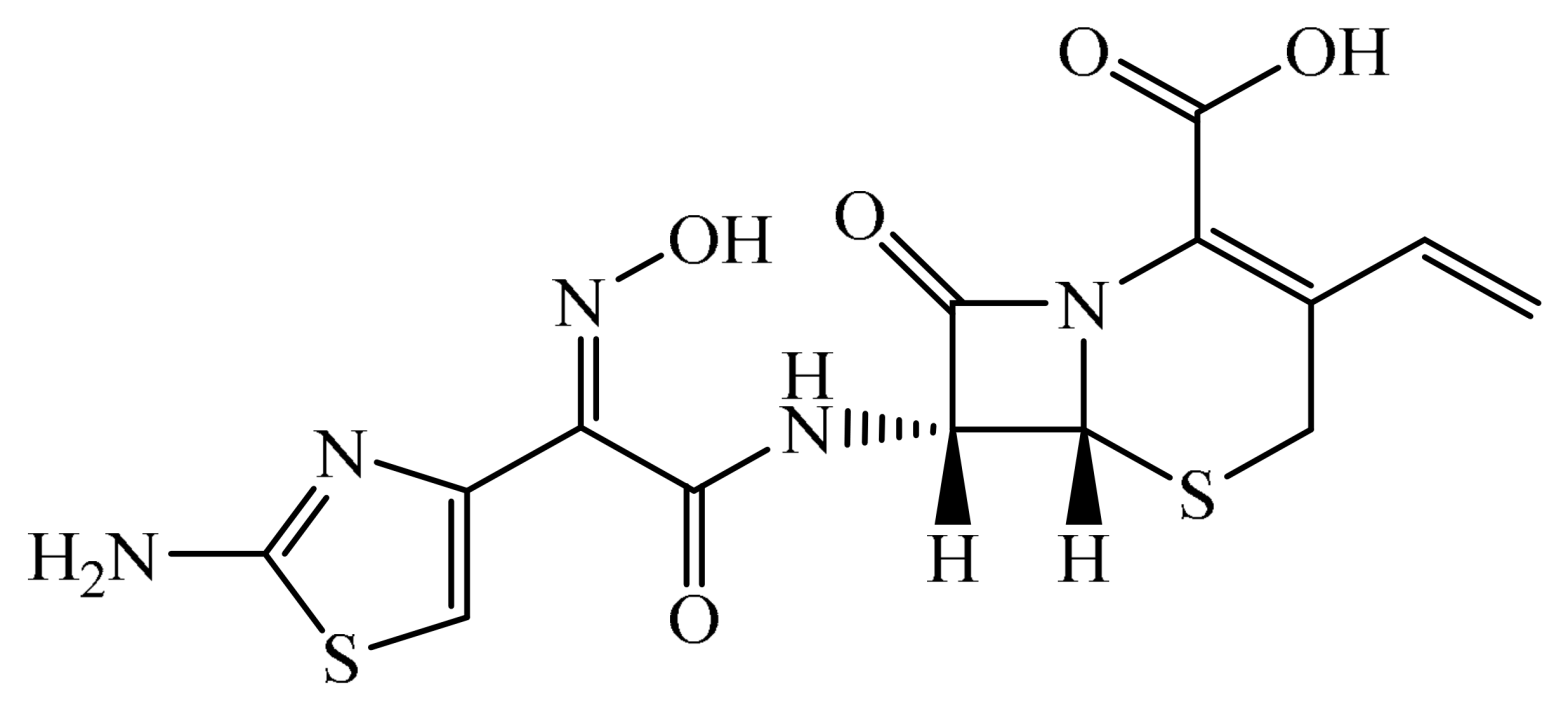	2.8	−1.8
	80210-62-4	Cefpodoxime	C_17_H_19_N_5_O_6_S_2_	453.5	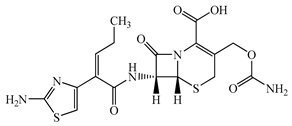	2.8 (Acid) 1.7 (Base)	0.4
	87239-81-4	Cefpodoxime proxetil	C_21_H_27_N_5_O_9_S_2_	557.6	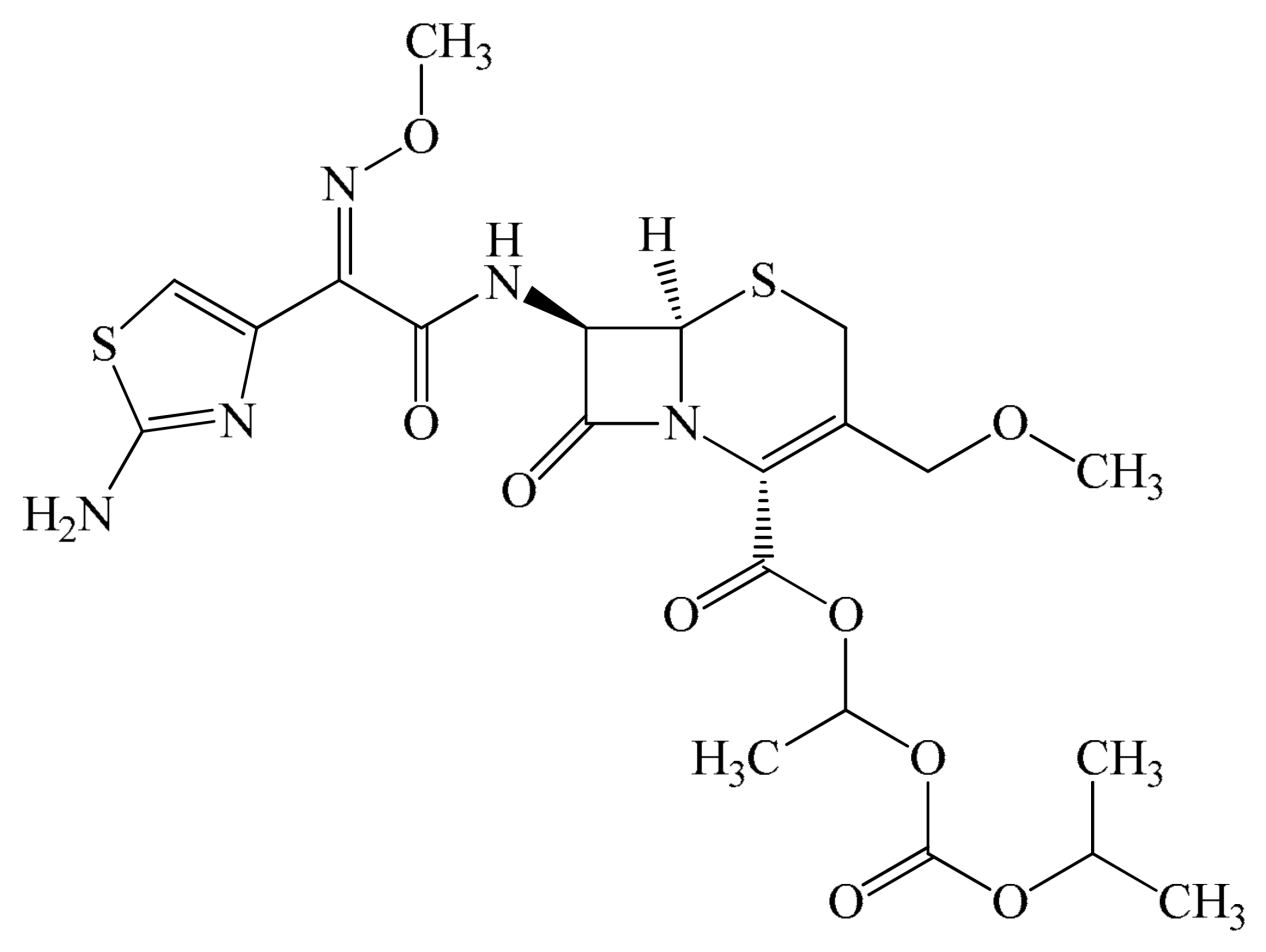	8.1 (Acid) 1.7 (Base)	2.9
	80370-57-6	Ceftiofur	C_19_H_17_N_5_O_7_S_3_	523.6	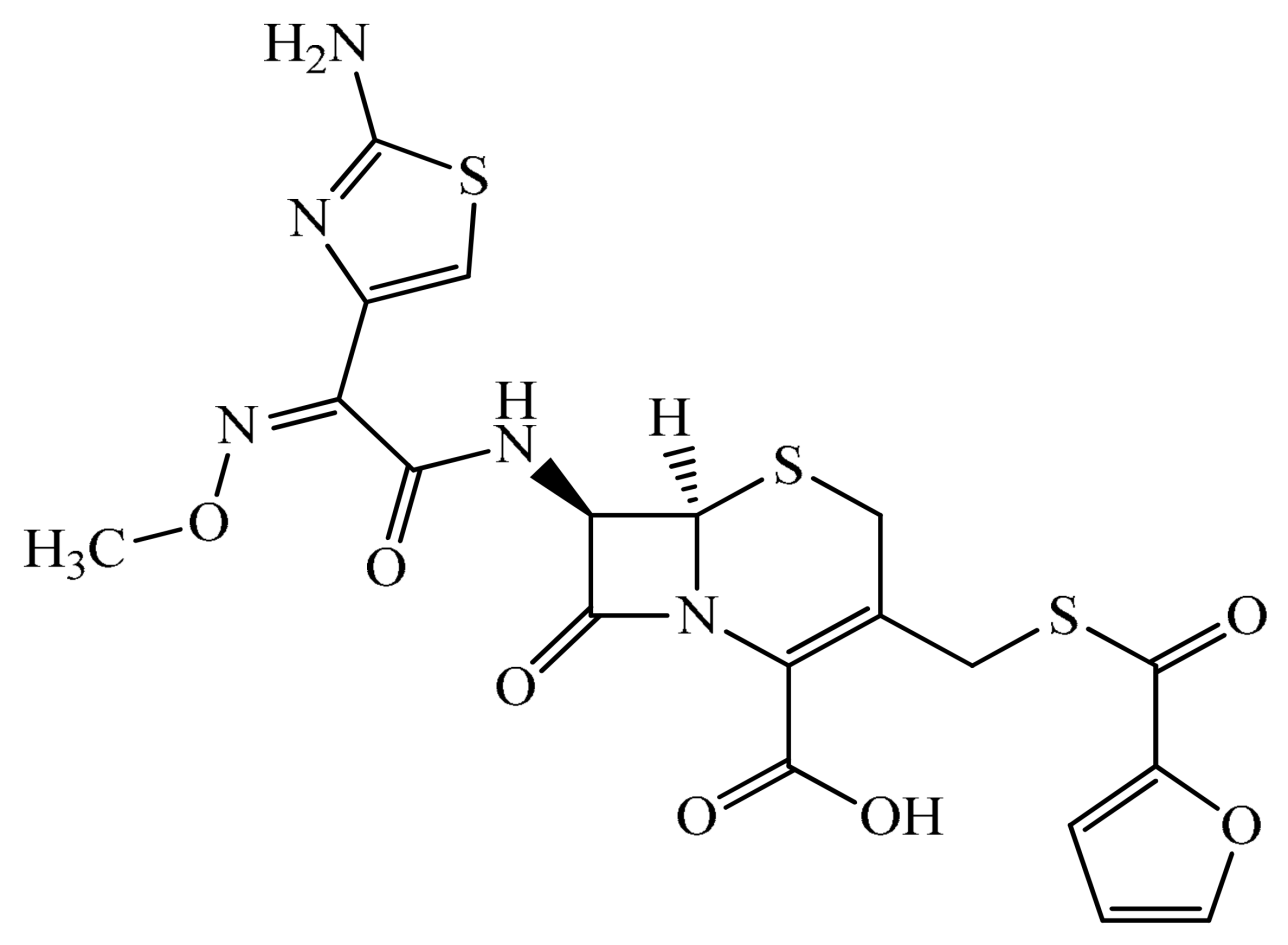	2.6	1.7
New quinolones	85721-33-1	Ciprofloxacin	C_17_H_18_FN_3_O_3_	331.3	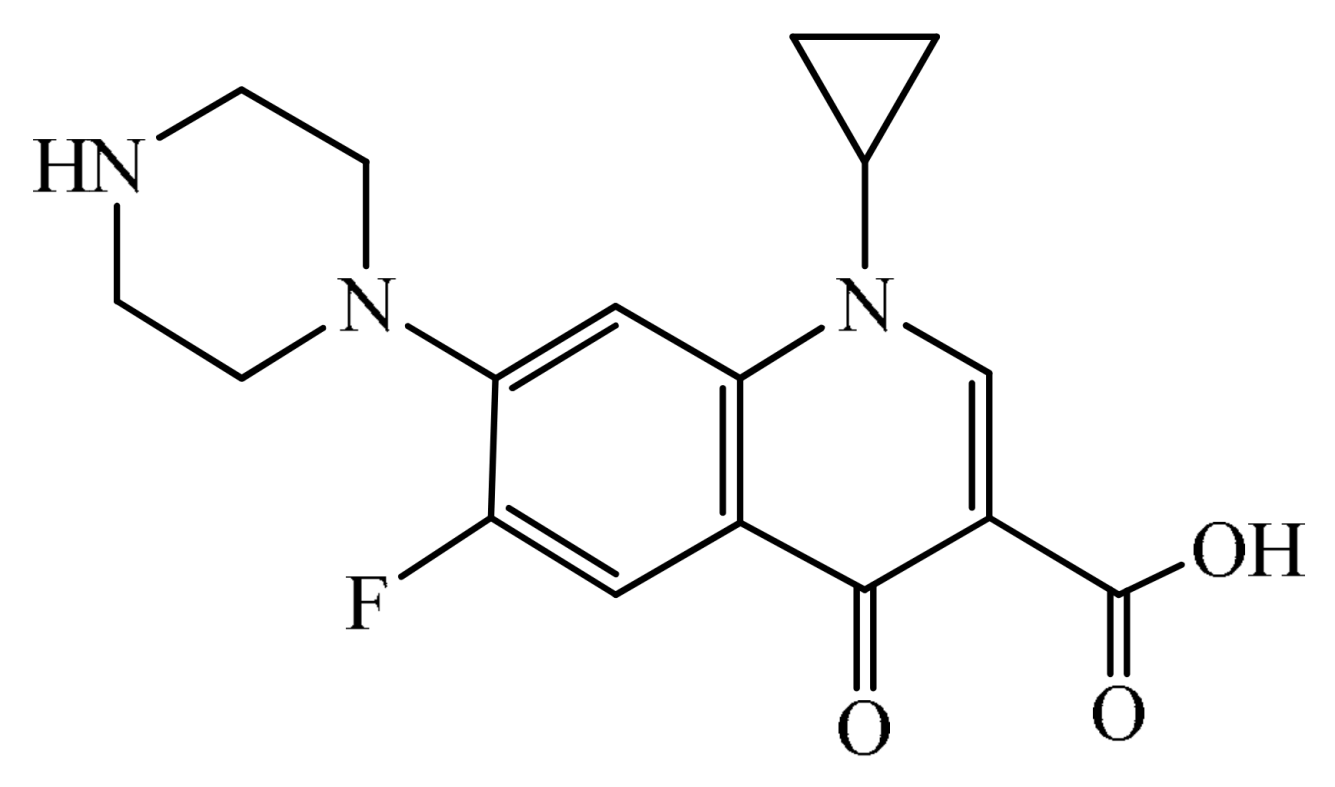	6.0	1.3
	93106-60-6	Enrofloxacin	C_19_H_22_FN_3_O_3_	359.4	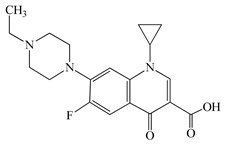	6.4	2.3
	100986-85-4	Levofloxacin	C_18_H_20_FN_3_O_4_	361.4	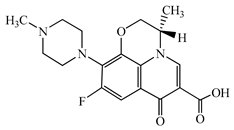	5.2	1.6
Macrolides	83905-01-5	Azithromycin	C_38_H_72_N_2_O_12_	749.0	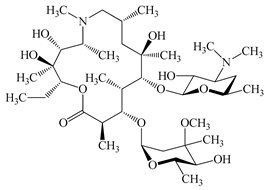	13.3	3.3
	81103-11-9	Clarithromycin	C_38_H_69_NO_13_	748.0	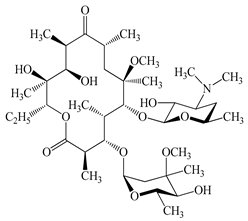	13.1	3.2
Tetracyclines	57-62-5	Chlortetracycline	C_22_H_23_ClN_2_O_8_	478.9	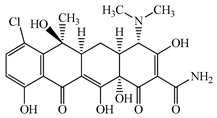	4.5	4.8
	564-25-0	Doxycycline	C_22_H_24_N_2_O_8_	444.4	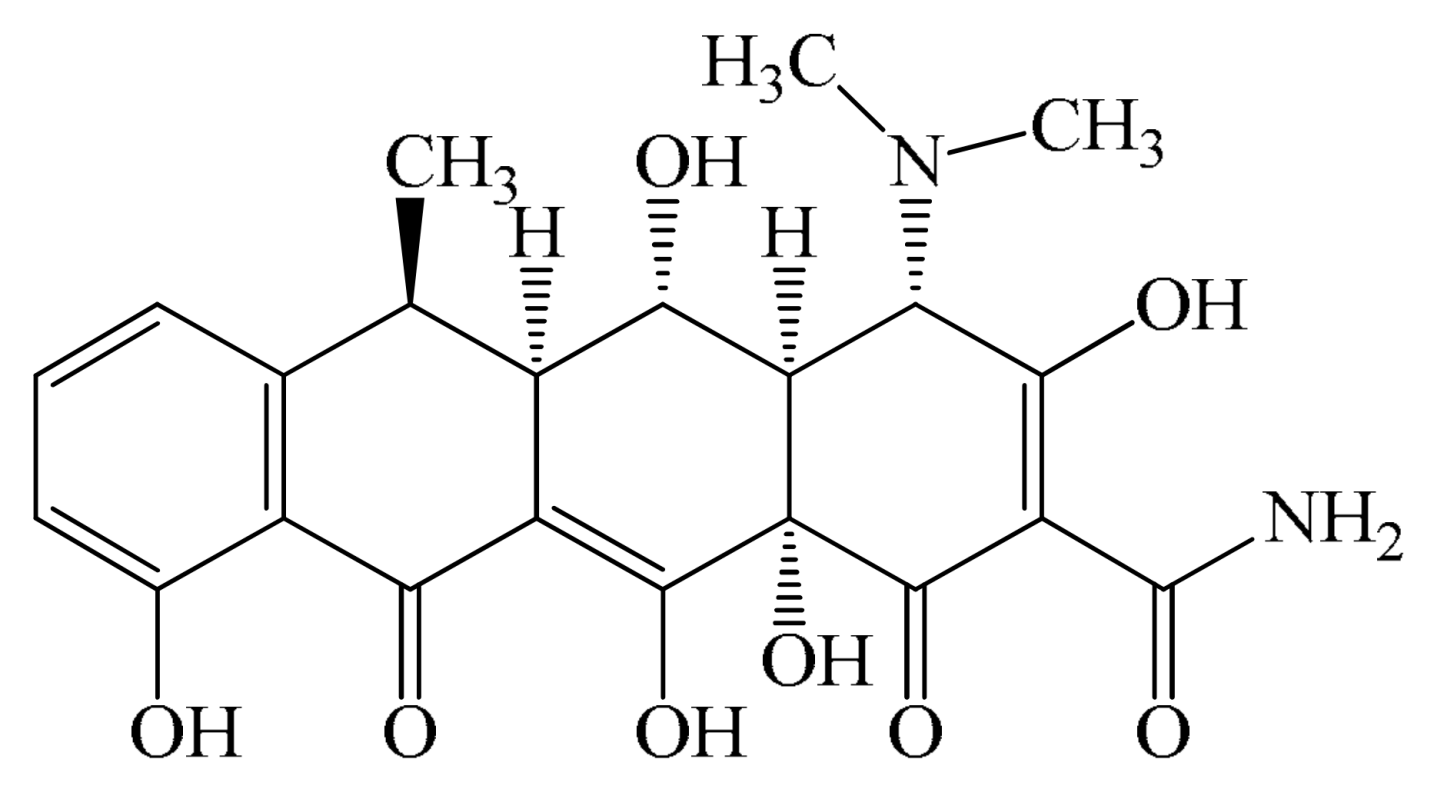	4.5 (Acid) 10.8 (Base)	1.8
	10118-90-8	Minocycline	C_23_H_27_N_3_O_7_	457.5	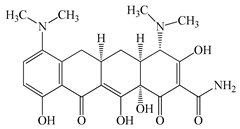	4.5 (Acid) 11.1 (Base)	2.2
	79-57-2	Oxytetracycline	C_22_H_24_N_2_O_9_	460.4	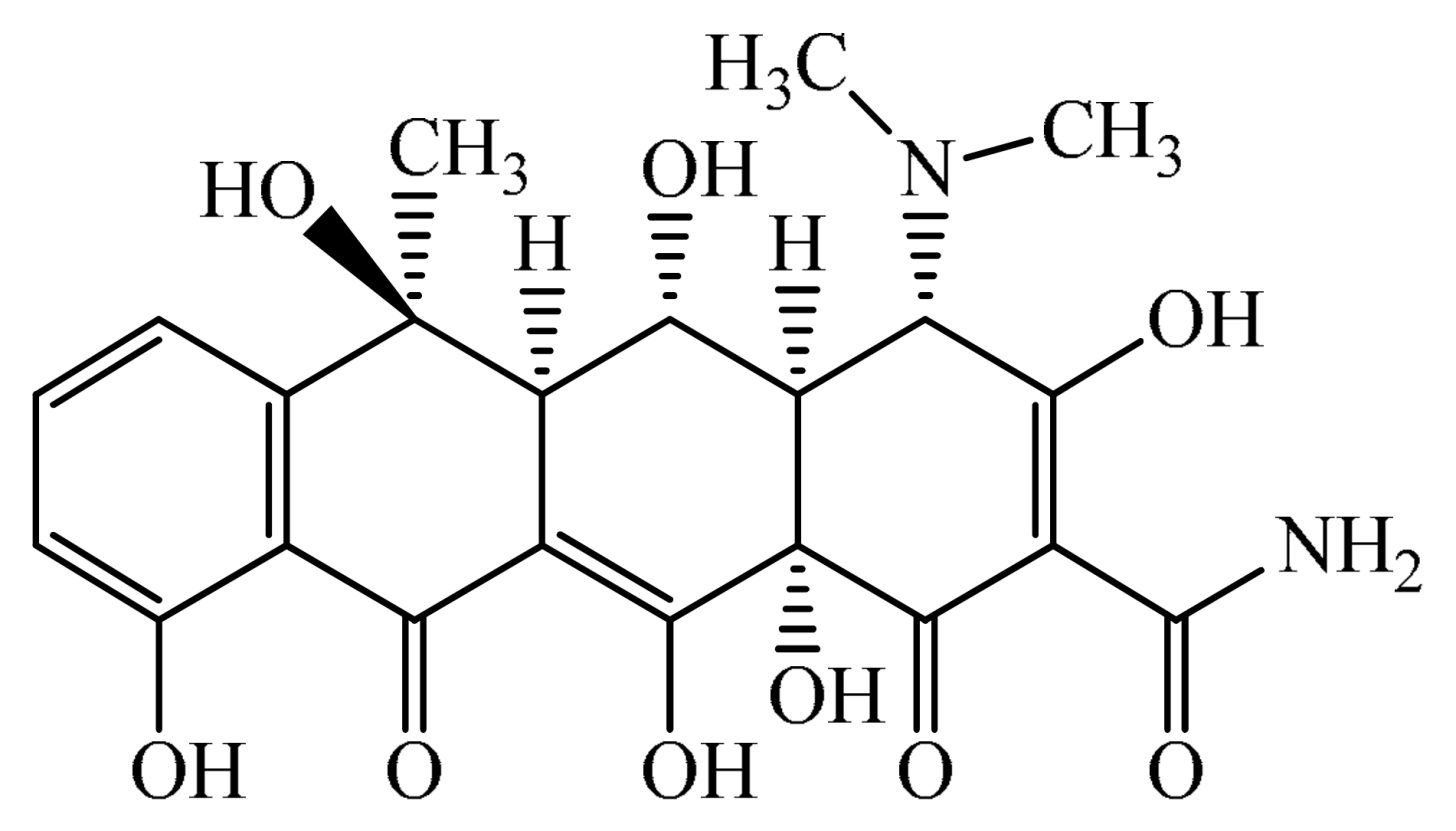	4.5	−1.5
	60-54-8	Tetracycline	C_22_H_24_N_2_O_8_	444.4	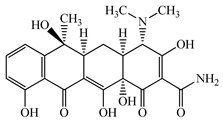	4.5	−1.5
Glycopeptide	1404-90-6	Vancomycin	C_66_H_75_Cl_2_N_9_O_24_	1449.3	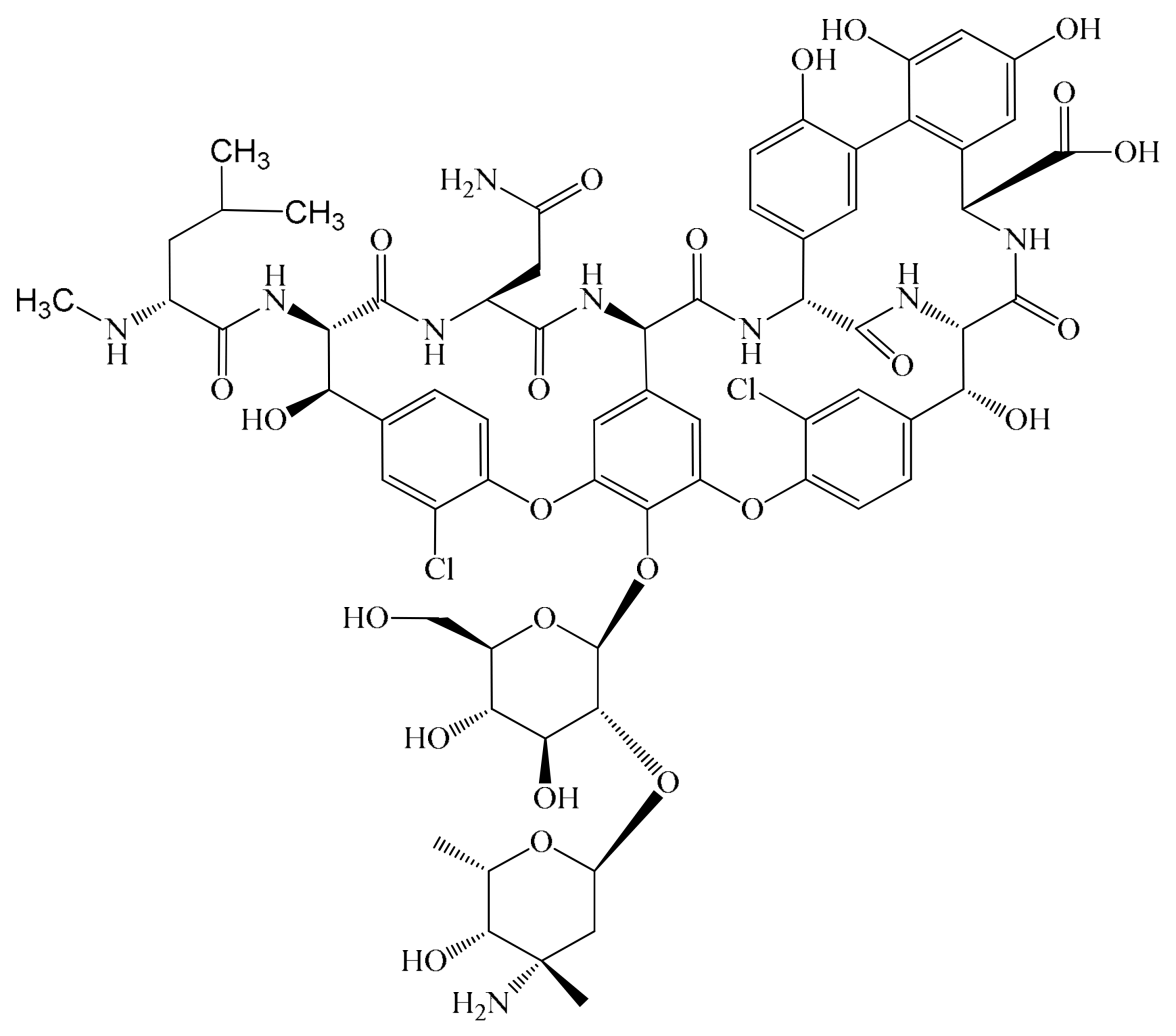	3.0	−1.4

CAS: Chemical Abstracts Service; p*K*_a_: acid dissociation constant; Log*P*: octanol-water partition coefficient.

**Table 2:** Detected concentrations of antimicrobials in hospital effluent, WWTP influent, WWTP secondary effluent, WWTP effluent, and river waters.

**Classification**	**Antimicrobial**	**Sample Type**											
		**River 1**				**River 2**				**WWTP Effluent**			
		**Concentration (ng/L)**				**Concentration (ng/L)**				**Concentration (ng/L)**			
		**Mean (SD)**		**Max**	**Min**	**Mean (SD)**		**Max**	**Min**	**Mean (SD)**		**Max**	**Min**
*β*-lactams	Ampicillin	15 (40)		105	N.D.	2 (5)		12	N.D.	412 (714)		1650	N.D.
	Benzylpenicillin	N.D.		N.D.	N.D.	N.D.		N.D.	N.D.	N.D.		N.D.	N.D.
	Cefdinir	N.D.		N.D.	N.D.	N.D.		N.D.	N.D.	N.D.		N.D.	N.D.
	Cefpodoxime	N.D.		N.D.	N.D.	N.D.		N.D.	N.D.	N.D.		N.D.	N.D.
	Cefpodoxime proxetil	N.D.		N.D.	N.D.	N.D.		N.D.	N.D.	N.D.		N.D.	N.D.
	Ceftiofur	N.D.		N.D.	N.D.	N.D.		N.D.	N.D.	N.D.		N.D.	N.D.
New quinolones	Ciprofloxacin	N.D.		N.D.	N.D.	N.D.		N.D.	N.D.	2 (5)		11	N.D.
	Enrofloxacin	N.D.		N.D.	N.D.	N.D.		N.D.	N.D.	N.D.		N.D.	N.D.
	Levofloxacin	6 (9)		24	N.D.	28 (33)		97	N.D.	539 (467)		1322	225
Macrolides	Azithromycin	N.D.		N.D.	N.D.	N.D.		N.D.	N.D.	113 (77)		196	N.D.
	Clarithromycin	12 (9)		28	N.D.	126 (124)		296	N.D.	497 (354)		913	N.D.
Tetracyclines	Chlortetracycline	N.D.		N.D.	N.D.	N.D.		N.D.	N.D.	N.D.		N.D.	N.D.
	Doxycycline	N.D.		N.D.	N.D.	N.D.		N.D.	N.D.	2 (3)		N.D.	N.D.
	Minocycline	N.D.		N.D.	N.D.	N.D.		N.D.	N.D.	N.D.		N.D.	N.D.
	Oxytetracycline	N.D.		N.D.	N.D.	N.D.		N.D.	N.D.	N.D.		N.D.	N.D.
	Tetracycline	N.D.		N.D.	N.D.	N.D.		N.D.	N.D.	N.D.		N.D.	N.D.
Glycopeptide	Vancomycin	N.D.		N.D.	N.D.	22 (32)		82	N.D.	42 (32)		72	N.D.
										
**Classification**	**Antimicrobial**	**Sample type**											
		**WWTP Secondary Effluent**			**WWTP Influent**			**Hospital Effluent 1**			**Hospital Effluent 2**		
		**Concentration (ng/L)**			**Concentration (ng/L)**			**Concentration (ng/L)**			**Concentration (ng/L)**		
		**Mean (SD)**	**Max**	**Min**	**Mean (SD)**	**Max**	**Min**	**Mean (SD)**	**Max**	**Min**	**Mean (SD)**	**Max**	**Min**
*β*-lactams	Ampicillin	89 (138)	314	N.D.	636 (741)	1878	N.D.	2589 (4237)	10,516	N.D.	3,723,848 (7,118,921)	18,580,444	N.D.
	Benzylpenicillin	N.D.	N.D.	N.D.	N.D.	N.D.	N.D.	N.D.	N.D.	N.D.	N.D.	N.D.	N.D.
	Cefdinir	N.D.	N.D.	N.D.	N.D.	N.D.	N.D.	3411 (9024)	23,875	N.D.	642 (1700)	4497	N.D.
	Cefpodoxime	N.D.	N.D.	N.D.	15 (39)	102	N.D.	8 (20)	54	N.D.	4047 (10,384)	27,590	N.D.
	Cefpodoxime proxetil	N.D.	N.D.	N.D.	N.D.	N.D.	N.D.	N.D.	N.D.	N.D.	N.D.	N.D.	N.D.
	Ceftiofur	N.D.	N.D.	N.D.	N.D.	N.D.	N.D.	N.D.	N.D.	N.D.	N.D.	N.D.	N.D.
New quinolones	Ciprofloxacin	1 (2)	4	N.D.	2 (6)	16	N.D.	5 (8)	20	N.D.	44 (89)	243	N.D.
	Enrofloxacin	N.D.	N.D.	N.D.	N.D.	N.D.	N.D.	N.D.	N.D.	N.D.	N.D.	N.D.	N.D.
	Levofloxacin	438 (356)	958	109	603 (354)	1150	87	556 (979)	2747	53	811 (834)	2606	250
Macrolides	Azithromycin	80 (70)	158	N.D.	411 (299)	729	N.D.	802 (1866)	5029	N.D.	3659 (8622)	23,166	N.D.
	Clarithromycin	433 (185)	651	165	669 (275)	1104	291	1122 (1723)	4398	40	4017 (9948)	26,560	N.D.
Tetracyclines	Chlortetracycline	N.D.	N.D.	N.D.	N.D.	N.D.	N.D.	15 (39)	102	N.D.	30 (79)	208	N.D.
	Doxycycline	5 (11)	25	N.D.	20 (53)	140	N.D.	N.D.	N.D.	N.D.	N.D.	N.D.	N.D.
	Minocycline	N.D.	N.D.	N.D.	27 (48)	118	N.D.	3631 (9594)	25,389	N.D.	107 (177)	468	N.D.
	Oxytetracycline	N.D.	N.D.	N.D.	N.D.	N.D.	N.D.	20 (53)	141	N.D.	N.D.	N.D.	N.D.
	Tetracycline	N.D.	N.D.	N.D.	N.D.	N.D.	N.D.	N.D.	N.D.	N.D.	N.D.	N.D.	N.D.
Glycopeptide	Vancomycin	118 (59)	191	44	183 (237)	714	39	36 (48)	114	N.D.	30,313 (66,190)	180,080	428

N.D.: Not Detected.

**Table 3:** Mass fluxes and contributions of antimicrobials in hospital effluent to WWTP influent.

Classification	Antimicrobial	Mean Mass Load (g/day)			Contribution of Hospital Effluent (% of Total WWTP Influent)		
		Hospital Effluent 1 Mean (SD)	Hospital Effluent 2 Mean (SD)	WWTP Influent Mean (SD)	Median	Max	Min
*β*-lactams	Ampicillin	0.27 (0.45)	1708 (3265)	104 (148)	4	89,408	N.A.
	Benzylpenicillin	N.D.	N.D.	N.D.	N.A.	N.A.	N.A.
	Cefdinir	0.36 (0.95)	0.29 (0.78)	N.D.	N.A.	N.A.	N.A.
	Cefpodoxime	N.D.	1.86 (4.76)	3 (7)	409	409	409
	Cefpodoxime proxetil	N.D.	N.D.	N.D.	N.A.	N.A.	N.A.
	Ceftiofur	N.D.	N.D.	N.D.	N.A.	N.A.	N.A.
New quinolones	Ciprofloxacin	N.D.	0.02 (0.04)	1 (1)	2	2	2
	Enrofloxacin	N.D.	N.D.	N.D.	N.A.	N.A.	N.A.
	Levofloxacin	0.05 (0.1)	0.31 (0.41)	93 (77)	2	6	N.A.
Macrolides	Azithromycin	0.08 (0.2)	1.68 (3.95)	61 (60)	2	82	N.A.
	Clarithromycin	0.12 (0.18)	1.84 (4.57)	106 (70)	3	37	N.A.
Tetracyclines	Chlortetracycline	N.D.	0.01 (0.04)	N.D.	N.A.	N.A.	N.A.
	Doxycycline	N.D.	N.D.	4 (10)	N.A.	N.A.	N.A.
	Minocycline	0.38 (1.01)	0.04 (0.08)	5 (9)	41	81	2
	Oxytetracycline	0.01 (0.01)	N.D.	N.D.	N.A.	N.A.	N.A.
	Tetracycline	N.D.	N.D.	N.D.	N.A.	N.A.	N.A.
Glycopeptide	Vancomycin	0.01 (0.01)	14 (30)	31 (47)	41	4478	5

N.D.: Not Detected, N.A.: Not Available. Values below the detection limit were excluded from the calculation of the contribution of mass fluxes. Some antimicrobials would not be detected in the influent at the wastewater treatment plant because of fluctuations in their concentration while flowing from the hospital through the sewer system.

**Table 4:** Mass fluxes and contributions of antimicrobials in WWTP effluent to river water.

Classification	Antimicrobial	Mean Mass Load (g/day)		Contribution of WWTP Effluent (% of Total River Water)		
		WWTP Effluent Mean (SD)	River 2 Mean (SD)	Median	Max	Min
*β*-lactams	Ampicillin	28 (43)	4 (10)	90	90	90
	Benzylpenicillin	N.D.	N.D.	N.A.	N.A.	N.A.
	Cefdinir	N.D.	N.D.	N.A.	N.A.	N.A.
	Cefpodoxime	N.D.	N.D.	N.A.	N.A.	N.A.
	Cefpodoxime proxetil	N.D.	N.D.	N.A.	N.A.	N.A.
	Ceftiofur	N.D.	N.D.	N.A.	N.A.	N.A.
New quinolones	Ciprofloxacin	N.D.	N.D.	N.A.	N.A.	N.A.
	Enrofloxacin	N.D.	N.D.	N.A.	N.A.	N.A.
	Levofloxacin	61 (67)	59 (76)	85	564	28
Macrolides	Azithromycin	11 (13)	N.D.	N.A.	N.A.	N.A.
	Clarithromycin	75 (46)	245 (295)	39	216	12
Tetracyclines	Chlortetracycline	N.D.	N.D.	N.A.	N.A.	N.A.
	Doxycycline	1 (2)	N.D.	N.A.	N.A.	N.A.
	Minocycline	N.D.	N.D.	N.A.	N.A.	N.A.
	Oxytetracycline	N.D.	N.D.	N.A.	N.A.	N.A.
	Tetracycline	N.D.	N.D.	N.A.	N.A.	N.A.
Glycopeptide	Vancomycin	18 (13)	38 (71)	10	44	0

N.D.: Not Detected, N.A.: Not Available. Values below the detection limit were excluded from the calculation of the contribution of mass fluxes.

## Data Availability

The data are not publicly available due to privacy or ethical restrictions. In conducting this study and publishing its results, an agreement with the hospital restricts disclosing any information that could lead to the identification of the hospital, including both information related to human subjects and the facility.

## Supplementary Materials

The following supporting information can be downloaded at: https://www.mdpi.com/article/10.3390/antibiotics15080760/s1, Table S1: LC-MS/MS parameters for the validation of each antimicrobial. The vertical axis shows the concentration of the antimicrobial on a logarithmic scale; Figure S1: Distribution of antimicrobials in hospital effluent, WWTP influent, WWTP secondary effluent, WWTP effluent, and river waters.
