# Supplementary material for: Mass Flux Analysis Reveals the Presence and Fate of Antimicrobials in Hospital Effluent, Wastewater Treatment Plant, and River Environments
Source: Antibiotics (Basel). 2026 Aug 7;15(8):760. doi: 10.3390/antibiotics15080760 (PMC13509301; doi:10.3390/antibiotics15080760)
Supplement: Supplementary file 1 [file antibiotics-15-00760-s001.zip › antibiotics-4467106-supplementary.pdf]

Supplemental Information for

Mass Flux Analysis Reveals the Presence and Fate of Antimicrobials in Hospital Effluent, Wastewater Treatment Plants, and River Environments

Author names: Takashi Azuma [1,2,*], Masaru Usui [3], Tomohiro Hasei [1], Tetsuya Hayashi [1]

Affiliation:
[1] Department of Pharmacy, Osaka Medical and Pharmaceutical University, Takatsuki 569-1094, Japan
[2] Center for Infectious Disease Education and Research (CiDER), The University of Osaka, Suita 565-0871, Japan
[3] Food Microbiology and Food Safety, Department of Health and Environmental Sciences, School of Veterinary Medicine, Rakuno Gakuen University, Ebetsu 069-8501, Japan

*Corresponding author: Takashi Azuma
Affiliation: Department of Pharmacy, Osaka Medical and Pharmaceutical University, 4-20-1 Nasahara, Takatsuki, Osaka 569-1094, Japan
Center for Infectious Disease Education and Research (CiDER), The University of Osaka, Suita 565-0871, Japan
Tel: +81-72-690-1055, Fax: +81-72-690-1055
e-mail address: takashi.azuma@ompu.ac.jp, t.azuma.mail@gmail.com

Table S1. LC-MS/MS parameters for the validation of each antimicrobial.

Classification	Compound	Ionization mode	Precursor ion (*m/z*)	Product ion (*m/z*)	Cone voltage (V)	Collision energy (eV)	Recovery (%) (SD)	LOD (ng/L)	LOQ (ng/L)
β-lactams	Ampicillin	ESI+	350.2	*105.9*, 192.0	29	24	101 (1)	3.2	10.7
	Benzylpenicillin	ESI+	335.2	*160.1*, 174.0	32	23	80 (8)	1.0	3.3
	Cefdinir	ESI+	369.2	170.0, *227.0*	30	20	111 (3)	0.5	1.6
	Cefpodoxime	ESI+	427.8	*240.5*, 395.9	31	15	97 (6)	0.6	2.1
	Cefpodoxime proxetil	ESI+	557.5	*409.8*, 525.2	30	18	112 (23)	2.4	7.9
	Ceftiofur	ESI+	524.1	*240.9*	38	18	88 (16)	1.3	4.3
New quinolones	Ciprofloxacin	ESI+	332.2	*288.2*, 314.2	40	25	58 (8)	1.3	4.5
	Enrofloxacin	ESI+	360.0	*316.2*, 245.2	37	19	61 (0)	0.5	1.5
	Levofloxacin	ESI+	362.2	261.2, *318.2*	40	21	61 (19)	0.6	2.0
Macrolides	Azithromycin	ESI+	749.8	*591.6*, 277.3	49	31	104 (15)	0.6	2.1
	Clarithromycin	ESI+	748.2	316.6, *558.3*	38	18	120 (24)	0.9	3.1
Tetracyclines	Chlortetracycline	ESI+	479.2	443.5, *461.5*	36	20	100 (4)	0.6	2.1
	Doxycycline	ESI+	445.2	*428.3*	32	18	95 (7)	0.6	2.0
	Minocycline	ESI+	458.3	*441.0*	36	21	122 (5)	0.6	2.0
	Oxytetracycline	ESI+	461.2	*425.8*	28	19	92 (12)	0.6	2.1
	Tetracycline	ESI+	445.2	*409.9*, 427.1	28	20	65 (15)	0.8	2.5
Glycopeptide	Vancomycin	ESI+	724.2	82.9, *100.2*	17	18	86 (5)	1.1	3.8

Product ions in italics were used for quantification

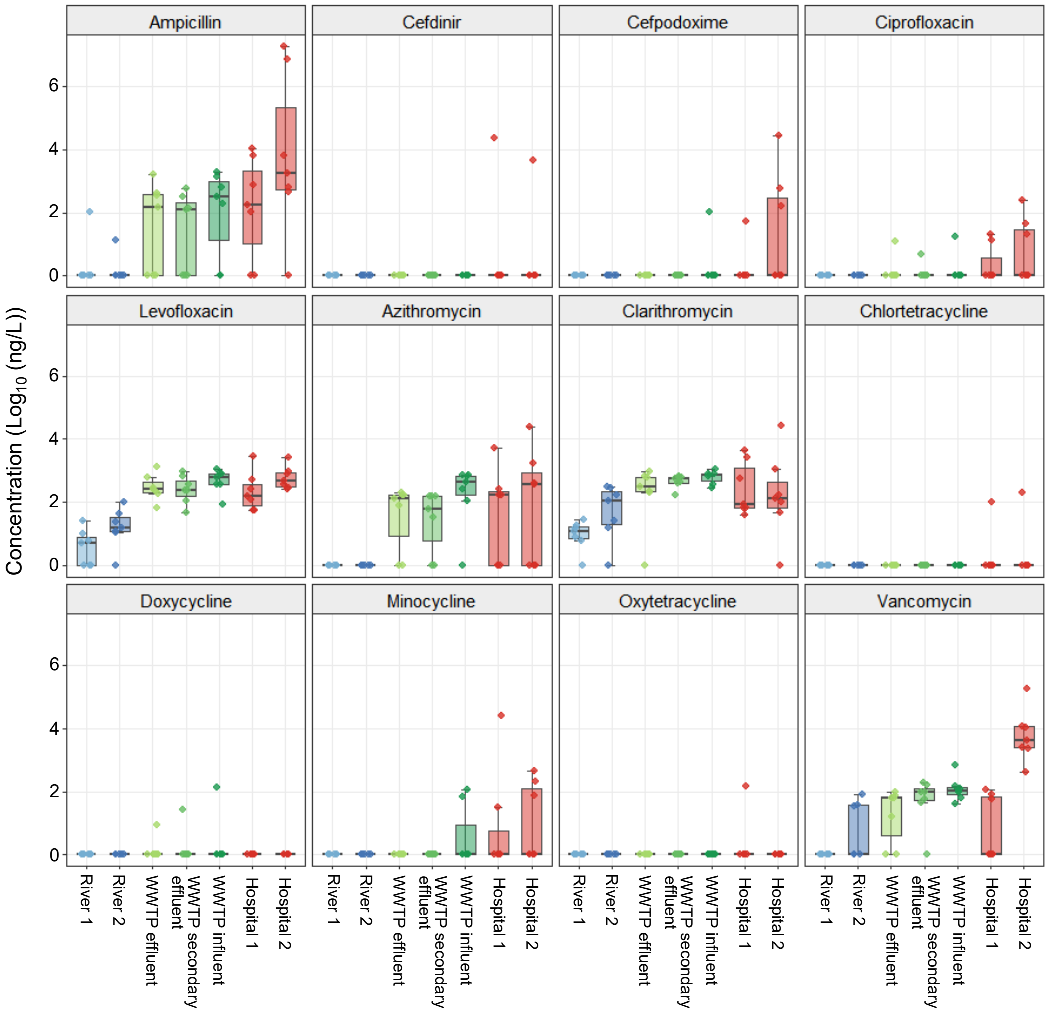

Figure S1. Distribution of antimicrobials in hospital effluent, WWTP influent, WWTP secondary effluent, WWTP effluent, and river waters. The vertical axis shows the concentration of the antimicrobial on a logarithmic scale.
